# Genome-wide identification and characterization of *auxin response factor* (*ARF*) family genes related to flower and fruit development in papaya (*Carica papaya* L.)

**DOI:** 10.1186/s12864-015-2182-0

**Published:** 2015-11-05

**Authors:** Kaidong Liu, Changchun Yuan, Haili Li, Wanhuang Lin, Yanjun Yang, Chenjia Shen, Xiaolin Zheng

**Affiliations:** College of Bioscience and Technology, Hunan Agricultural University, Changsha, Hunan 410128 China; College of Food Science and Biotechnology, Zhejiang Gongshang University, Hangzhou, 310035 China; College of Life and Environmental Sciences, Hangzhou Normal University, Hangzhou, 310036 China; Life Science and Technology School, Lingnan Normal University, Zhanjiang, Guangdong 524048 China

**Keywords:** Auxin, Auxin response factor, Papaya, Developmental process, Fruit ripening

## Abstract

**Background:**

Auxin and auxin signaling are involved in a series of developmental processes in plants. Auxin Response Factors (ARFs) is reported to modulate the expression of target genes by binding to auxin response elements (AuxREs) and influence the transcriptional activation of down-stream target genes. However, how ARF genes function in flower development and fruit ripening of papaya (*Carica papaya* L.) is largely unknown. In this study, a comprehensive characterization and expression profiling analysis of 11 *C. papaya ARF* (*CpARF*) genes was performed using the newly updated papaya reference genome data.

**Results:**

We analyzed *CpARF e*xpression patterns at different developmental stages. *CpARF1*, *CpARF2*, *CpARF4*, *CpARF5*, and *CpARF10* showed the highest expression at the initial stage of flower development, but decreased during the following developmental stages. *CpARF6* expression increased during the developmental process and reached its peak level at the final stage of flower development. The expression of *CpARF1* increased significantly during the fruit ripening stages. Many AuxREs were included in the promoters of two ethylene signaling genes (*CpETR1* and *CpETR2*) and three ethylene-synthesis-related genes (*CpACS1*, *CpACS2*, and *CpACO1*), suggesting that CpARFs might be involved in fruit ripening via the regulation of ethylene signaling.

**Conclusions:**

Our study provided comprehensive information on *ARF* family in papaya, including gene structures, chromosome locations, phylogenetic relationships, and expression patterns. The involvement of *CpARF* gene expression changes in flower and fruit development allowed us to understand the role of ARF-mediated auxin signaling in the maturation of reproductive organs in papaya.

**Electronic supplementary material:**

The online version of this article (doi:10.1186/s12864-015-2182-0) contains supplementary material, which is available to authorized users.

## Background

Auxin is a plant hormone that plays pivotal roles in the regulation of plant growth in response to diverse developmental and environmental events such as embryogenesis, organogenesis, tropic growth, root architecture, flower and fruit development, tissue and organ patterning, and vascular development [1–3]. It has been shown that auxin coordinates plant development essentially through the transcriptional regulation of some gene families, such as auxin/indole-3-acetic acid (*Aux/IAA*), Gretchen Hagen3 (*GH3*), small auxin up RNA (*SAUR*), and auxin response factor (*ARF*) [4, 5]. It was subsequently found that these so-called early auxin-responsive genes are characterized by conserved promoter elements, including the TGA element (AACGAC), core element of the auxin response region (AuxRE-core; GGTCCAT), and auxin response element (AuxRE; TGTCTC) [6, 7]. Being an important component of auxin signaling pathway, ARFs activate or repress the expression of auxin response genes by binding to AuxRE in their promoter [8].

A typical ARF contains a highly conserved N-terminal B3-like DNA binding domain (DBD) that recognizes AuxRE in the promoter of auxin-responsive genes [8]. The C-terminal dimerization domain (CTD) contains two motifs, called III and IV, that are also found in Aux/IAA and enable the formation of homo- and hetero-dimers among ARFs and Aux/IAAs [9, 10]. The middle region (MR), located between DBD and CTD, confers transcriptional activation or repression depending on its amino acid composition [8, 11].

The functions of *ARFs* are well studied. In *Arabidopsis thaliana*, *arf1* and *arf2* loss-of-function mutations affect leaf senescence and floral organ abscission [12]. Loss-of-function *arf3* mutants display defects in gynoecium and floral meristem patterning [13, 14], while mutant *arf5* is characterized by abnormal vascular strands and embryo axis [15]. AtARF7 is involved in the conditional regulation of differential growth in aerial tissues, and a mutation in *AtARF7* impairs hypocotyl response to blue light and auxin stimuli [16]. AtARF8 regulates hypocotyl elongation, auxin homeostasis, and fruit development [12, 17]. Furthermore, the flowers of *arf6/arf8* double mutant are infertile closed buds with short petals, short stamen filaments, and undehisced anthers [18]. The double mutation, *arf7/arf19* affects auxin mediated lateral root development [19]. In rice (*Oryza sativa* L.), transgenic plants that express an antisense *OsARF1* show extremely low growth, poor vigor, curled leaves, and sterility, suggesting that this gene is essential for vegetative and reproductive development [20]. Previous studies have shown that OsARF16, a transcription factor regulating auxin redistribution, is required for iron and phosphate deficiency responses in rice [21–23]. Another auxin response factor, OsARF19, controls rice leaf angles through the positive regulation of OsGH3–5 and OsBRI1 [24]. In tomato (*Solanum lycopersicon*), recent studies have shown the involvement of *SlARF* genes in flower development and fruit set, development, and ripening [25–27].

Papaya (*Carica papaya* L.) is an economically important fruit crop in tropical and subtropical countries [28]. Sex type in this trioecious species is determined by a pair of sex chromosomes, and plants have either female (XX), male (XY), or hermaphrodite [XY(h)] flowers [29]. Papaya often exhibits male and imperfect hermaphrodite flowers, which are influenced by environmental and hormonal factors [30–32]. Under high summer temperatures, the flowers have been observed to change from hermaphrodite to male because of ovary abortion and stamen carpelloid. Some endohormones, such as auxin, may play important roles in this change process [28, 33]. Despite the various causes of malformation in papaya fruit, the pear-shaped fruits from hermaphrodite flowers are commercially preferred, and hermaphrodite papayas are favored worldwide for economic production [28, 34]. Papaya fruits are very susceptible to deterioration and postharvest losses mainly by fungal decay and physiological disorders such as chilling injury, pests, mechanical injury, and over-ripeness. Therefore, there are several critical problems in breeding and cultivation of hermaphrodite plants that need to be solved [35]. Auxin has a positive role in the quality maintenance and shelf life of harvested papaya fruits [36]. Application of exogenous auxin can delay fruit ripening in many crop species [34]; however, the underlying mechanism linking auxin signaling and reproduction of papaya is largely unknown.

As an important segment of the auxin-signaling pathway, ARFs are encoded by a multi-gene family in many different plant species. There are 23 members in *Arabidopsis*, 22 in tomato, 31 in maize (*Zea mays* L.), 15 in cucumber (*Cucumis sativus*), 39 in poplar (*Populus trichocarpa*), 25 in rice (*Oryza sativa* L.), 24 in Medicago (*Medicago truncatula*), 19 in sweet orange (*Citrus sinensis*), and 51 in soybean (*Glycine max* L.) [5, 7, 21, 37–42]. In this study, we used the existing data in public databases to perform domain analysis and identify genes encoding ARFs in papaya. We also aimed to reveal comprehensive information on the gene structure, protein motif architecture, and sequence homology of 11 CpARFs.

## Results

### Genome-wide identification of *CpARF* genes

A total of 11 *ARF*s were identified in *C. papaya*. These genes were named according to the phylogenetic relationships between *C. papaya* and *Arabidopsis*. Comprehensive information on these 11 *CpARF* genes, including gene name, locus ID, open reading frame (ORF) length, number of introns, location on supercontigs and deduced polypeptide sequences, is presented in Table 1. The size of deduced CpARFs ranged from 311 (CpARF6) to 938 amino acids (CpARF5), the corresponding molecular masses from 34.83 to 103.7 kDa, and the predicted isoelectric points from 5.16 (CpARF5) to 9.03 (CpARF6). All the nucleic acid sequences were listed in the Additional file 1: Table S1.

**Table 1:** The information of ARF family genes in *Carica papaya* ^*a*^

						Deduced polypeptide		
Gene ID	Name^b^	Location^c^	Direction	ORF length	Introns	Length (aa)	Mol wt (kDa)	pI
evm. TU.supercontig_9.161	CpARF1	supercontig_9:969763..974848	Reverse	2094	13	698	77.67	7.18
evm.TU.contig_31756.1	CpARF2	contig_31756:3939..7439	Forward	1855	11	619	68.86	7.12
evm.TU.supercontig_7.3	CpARF3	supercontig_7:132322..138926	Reverse	2022	10	674	73.19	7.01
evm.TU.supercontig_139.80	CpARF4	supercontig_139:638531..645762	Reverse	2439	11	813	89.78	6.58
evm.TU.supercontig_26.24	CpARF5	supercontig_26:231561..267729	Reverse	2814	13	938	103.7	5.16
evm.TU.supercontig_17.53	CpARF6	supercontig_17:617715..620541	Reverse	933	8	311	34.83	9.03
evm.TU.supercontig_261.2	CpARF7	supercontig_261:2520..11208	Reverse	2649	12	883	97.65	5.52
evm.TU.supercontig_65.4	CpARF10	supercontig_65:11160..14085	Reverse	1944	4	648	71.54	7.06
evm.TU.supercontig_96.40	CpARF11	supercontig_96:684489..688508	Forward	2064	13	688	76.05	6.66
evm.TU.supercontig_53.88	CpARF16	supercontig_53:584129..586644	Reverse	2091	2	697	76.94	6.57
evm.TU.supercontig_49.122	CpARF17	supercontig_49:862531..867248	Reverse	1809	1	603	66.22	6.51

^a^The information listed in table was obtained from Phytozome 10.1

^b^Names of ARF genes in *Carica papaya* were based on the nomenclature used in the *Arabidopsis* model species

^c^The location of different CpARF genes on each contig or supercontig

### Analysis of phylogenetic relationships and gene structure

**Fig. 1:**
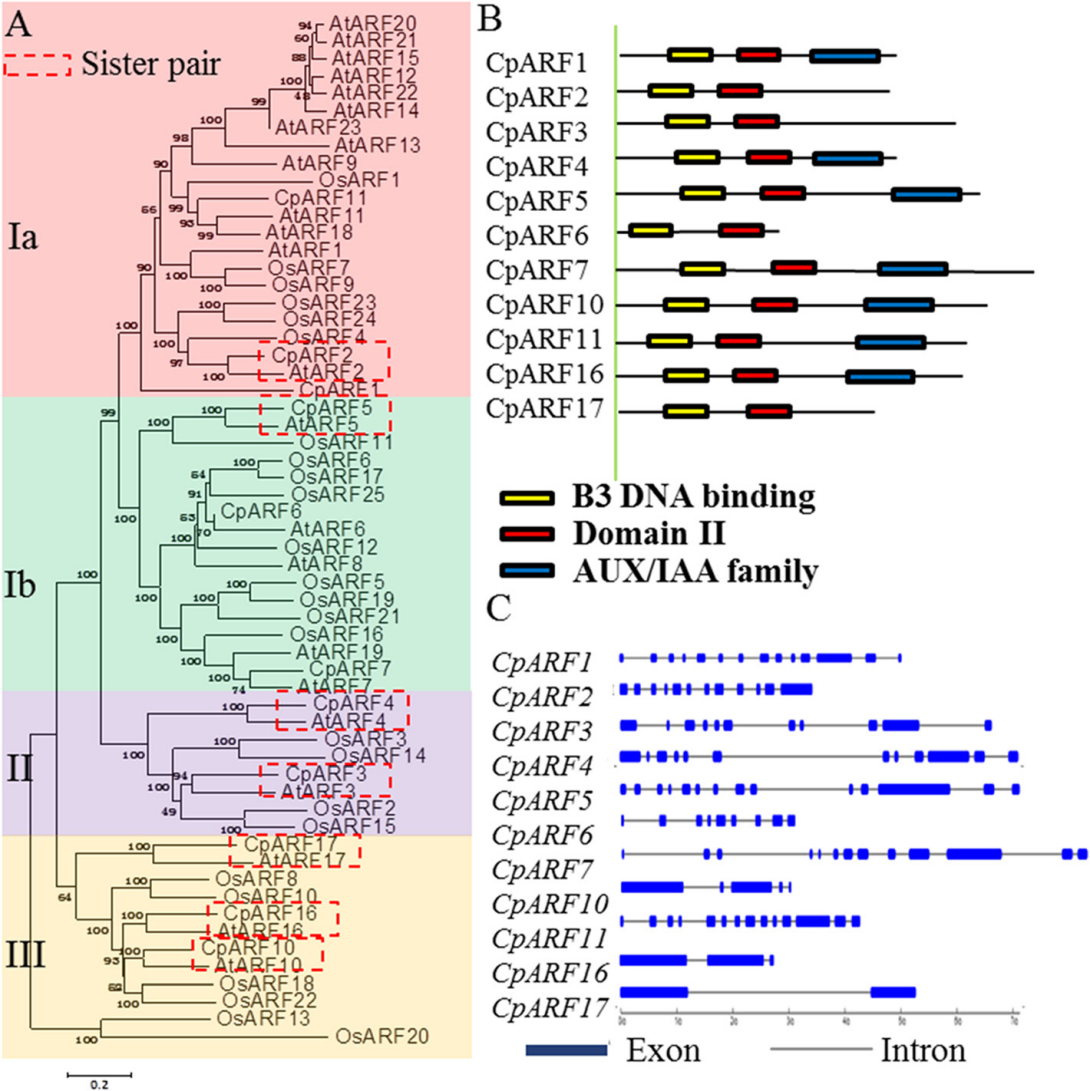
Analysis of protein domains, gene structures, and phylogenesis. **a** Phylogeny of auxin response factor (ARF) proteins between different species. Eleven *Carica papaya* ARFs (CpARFs), 23 *Arabidopsis thaliana* ARFs (AtARFs), and 25 *Oryza sativa* ARFs (OsARFs) are classified into three groups: I, II, and III. Group I includes two subgroups: Ia and Ib. Scale bar 0.1 denotes 0.1 amino-acid substitution per site. **b** Schematic organization of CpARFs. The ARF domain, B3 DNA binding domain (DBD), and auxin/indole-3-acetic acid (AUX/IAA) family domain are shown in red, yellow, and blue, respectively. **c** Exon-intron structure analysis of CpARF genes. Exons are represented by blue boxes; introns are represented by gray lines

The phylogenetic distribution suggested that *ARF*s could be grouped into four major subclasses, including Ia, Ib, II, and III (Fig. 1a). Based on the phylogenetic tree, seven sister gene pairs were identified between *Arabidopsis* and *C. papaya*: *CpARF2*/*AtARF2*, *CpARF3*/*AtARF3*, *CpARF4*/*AtARF4*, *CpARF5*/*AtARF5*, *CpARF10*/*AtARF10*, *CpARF16*/*AtARF16*, and *CpARF17*/*AtARF17*. No sister gene pairs were found between *C. papaya* and rice. Most CpARFs contained three typical domains: DBD, domain II, and AUX/IAA family domain. CpARF2, CpARF3, CpARF6, and CpARF17 contained DBD and domain II, but no AUX/IAA family domain (Fig. 1b). The exon-intron structure of each *CpARF* was revealed by comparing the full-length cDNA sequences with the corresponding genomic DNA sequences. The number of introns in *CpARF* genes ranged from 1 to 13 (Fig. 1c). *CpARF* genes, even with close phylogenetic relationship, displayed complex distribution patterns of introns-exons.

### Analysis of amino-acid composition and classification of CpARFs

**Fig. 2:**
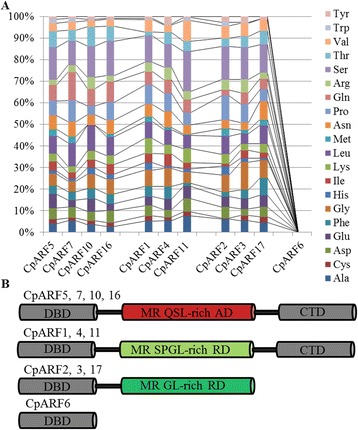
Analysis of amino acid content and classification of *Carica papaya* auxin response factor (CpARF) proteins. **a** The protein structure of CpARFs. DBD, DNA-binding domain; CTD, C-terminal dimerization domain; MR, middle region; RD, repression domain; AD, activation domain; Q, glutamine; S, serine; L, leucine; P, proline; G, glycine. **b** Amino-acid content of MR domains in putative CpARFs. CpARF is the X-axis variable and the corresponding amino acid content is the Y-axis variable. Colored bars represent different amino acids

The 11 CpARFs were classified into three groups based on their MR amino-acid composition and the presence or absence of CTDs: (1) ARFs with a DBD, activator MR and a CTD; (2) ARF with a DBD, repressor MR and a CTD; and (3) ARFs with a DBD, repressor MR, but no CTD (Fig. 2a and Additional file 2: Figure S1). The domain position in these 11 CpARFs is presented in Additional file 3: Table S2, and the amino acid composition of MRs is shown in Fig. 2b and Additional file 4: Table S3. CpARFs contained four putative transcriptional activators, CpARF5, seven, ten, and 16 (QSL-rich MR), and three putative transcriptional repressors, CpARF1, four, and 11 (SLPG-rich MR). Three CpARFs (CpARF2, three, and 17) were putative transcriptional repressors that did not contain a CTD. Only one CpARF, CpARF6, contained only a DBD.

### Expression patterns for *CpARF* genes in different plant tissues

**Fig. 3:**
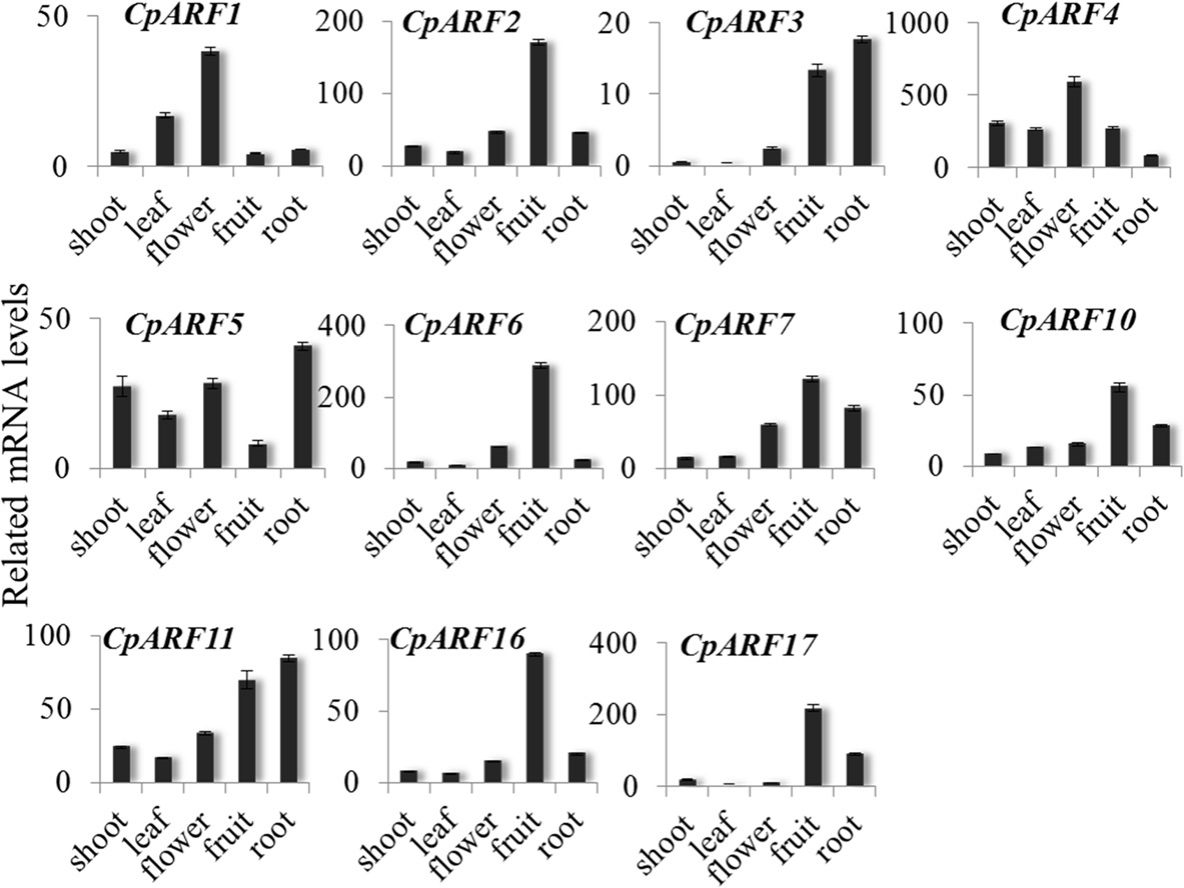
Tissue-specific expression patterns of *Carica papaya* auxin response factor (CpARF) genes. Expression pattern of CpARF genes in leaves, shoots, roots, flowers, and fruits of 2-year-old papaya plants. *CpACTIN* value is 1000. Means are from five independent repeats; error bars show standard deviations

To study the physiological function of CpARF genes, the spatial-specific expression pattern of the 11 CpARF genes was detected in different tissues and organs, including shoots, leaves, flowers, fruits and roots. The expression of most CpARF genes was ubiquitous in all studied tissues and organs, suggesting that they might have a putative function in many aspects of plant growth and development. Some CpARF genes (*CpARF2*, *CpARF6*, *CpARF10*, *CpARF16*, and *CpARF17*) showed fruit-specific expression, which indicated that they might play a role in fruit ripening. *CpARF1* was highly expressed in flowers, while *CpARF3*, *CpARF5*, and *CpARF11* were highly expressed in roots. Many CpARF genes, including *CpARF1*, *CpARF2*, *CpARF3*, *CpARF6*, *CpARF7*, *CpARF16*, and *CpARF17*, were hardly detectable in leaves and shoots (Fig. 3).

### Expression of *CpARF* genes during flower developmental stages and fruit set

**Fig. 4:**
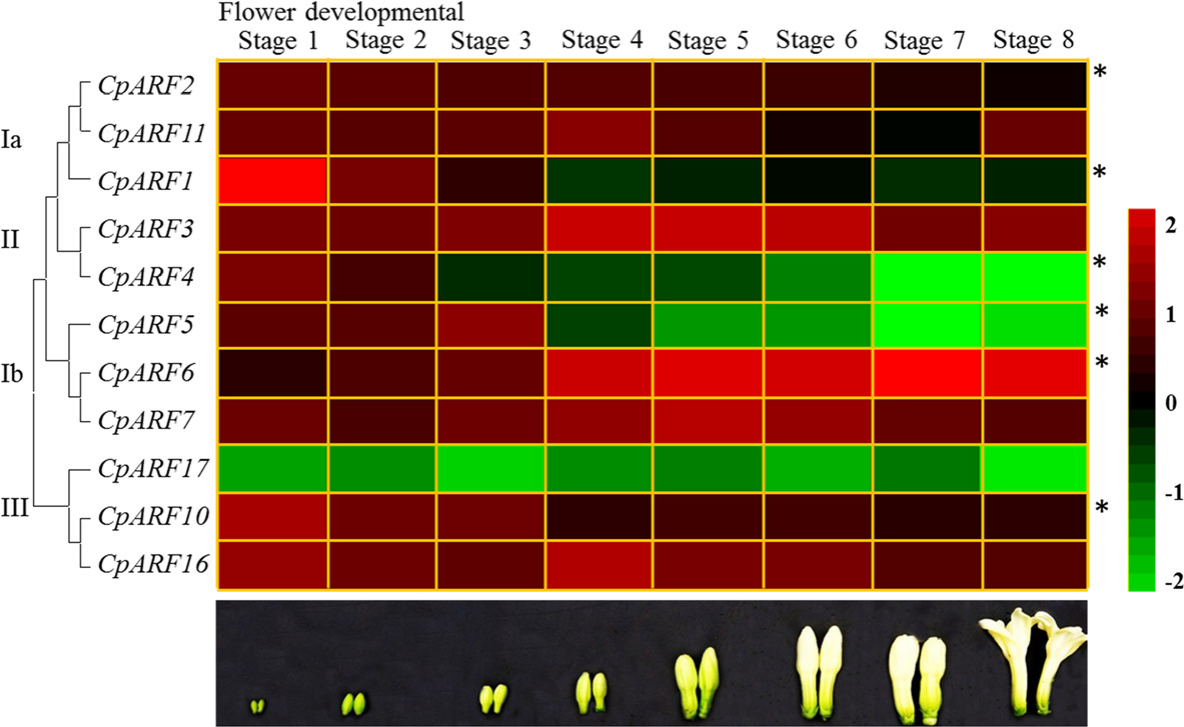
Heatmap of *Carica papaya* auxin response factor (CpARF) gene expression during different flower developmental stages. Changes in the expression levels during different flower developmental stages that schematically depicted above the displayed quantitative real time (qRT) data are relative to RNA accumulation levels. Levels of down expression (*green*) or up expression (*red*) are shown on a log2 scale from the highest to the lowest expression of each CpARF gene. Significant (*P* < 0.05) differences are indicated by an asterisk

In our study, we focused on the expression pattern of CpARF genes in flowers during eight different developmental stages. Except for *CpARF17* that showed the lowest expression level in all flowering stages, the remaining *CpARF* genes exhibited dynamic expression patterns. *CpARF1*, *CpARF2*, *CpARF4*, *CpARF5*, and *CpARF10* showed the peak expression in flower developmental stage one and decreased during the following developmental stages, while *CpARF6* increased during the developmental process and reached the peak at stage seven. In addition, the expression pattern of CpARF genes that belonged to the same phylogenetic branch also varied significantly. The expression of *CpARF3* did not change significantly during the developmental process, while the expression of its sister pair gene, *CpARF4*, showed a clear decrease (Fig. 4 and Additional file 5: Table S4).

**Fig. 5:**
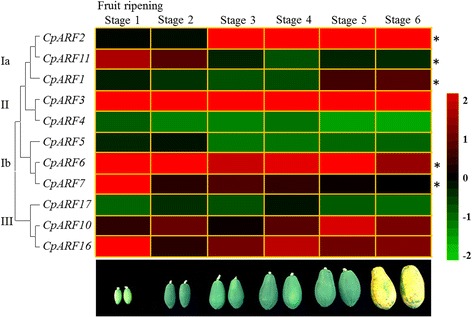
Heatmap of *Carica papaya* auxin response factor (CpARF) gene expression during different fruit developmental stages. Changes in the expression levels during different fruit developmental stages that schematically depicted above the displayed quantitative real time (qRT) data are relative to RNA accumulation levels. Levels of down expression (*green*) or up expression (*red*) are shown on a log2 scale from the highest to the lowest expression of each CpARF gene. Significant (*P* < 0.05) differences are indicated by an asterisk

Tissue-specific expression analysis showed that some CpARF genes were highly expressed in the reproductive organs (Fig. 3). These results prompted us to investigate the expression of CpARF genes during various fruit ripening stages. The data indicated that the expression of most CpARF genes underwent a significant change associated with fruit ripening. The expression of *CpARF1* showed a significant increase during the fruit ripening stages; while the expression of *CpARF7* and *CpARF11* decreased from stage one to stage six (Fig. 5 and Additional file 6: Table S5).

### Auxin regulation of *CpARF* genes in the flower and fruit

The qRT-PCR data showed that most of CpARF genes were responsive to IAA and TIBA treatment.

In flowers, the expression of *CpARF1* was significantly down regulated by IAA treatment and up regulated by TIBA treatment. *CpARF2* and *CpARF3* expression levels were significantly increased by IAA treatment and remained stable after TIBA treatment. *CpARF6* showed no response to IAA treatment and was largely induced by TIBA treatment. *CpARF10* also showed no response to IAA treatment and was significantly induced by TIBA treatment. *CpARF5* showed opposite expression patterns between IAA treatment and TIBA treatment. The expression of *CpARF5* was up regulated by IAA treatment and down regulated by TIBA treatment (Additional file 7: Figure S2).

In fruits, many CpARF genes, such as *CpARF1*, *CpARF4*, C*pARF5*, *CpARF7*, *CPARF11*, and *CpARF16*, were significantly induced by TIBA treatment. However, many CpARF genes, including *CpARF2*, *CpARF4*, *CpARF6*, *CpARF10*, *CpARF16*, and *CpARF17*, were inhibited by IAA treatment (Additional file 8: Figure S3).

### Expression of C*pARF* genes involved in male-hermaphrodite differentiation

**Fig. 6:**
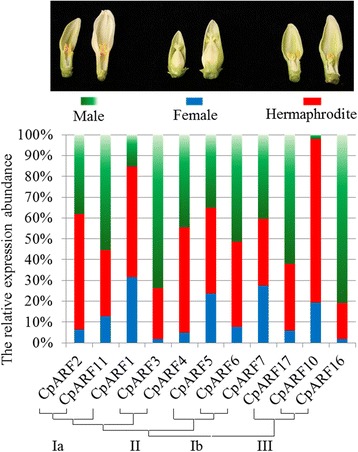
Expression of *Carica papaya* auxin response factor (CpARF) genes in different flower sex types. Three different sex type flowers were collected for qRT-PCR test. The green boxes indicated the expression abundance in male flowers; the blue boxes indicated the expression abundance in female flowers; the red boxes indicated the expression abundance in hermaphrodite flowers

To understand the regulatory mechanisms of auxin signaling involved in sex determination, we analyzed the expression abundance of CpARF genes in the three different sex types. Most CpARF genes showed higher expression abundance in male and hermaphrodite flowers than in female flowers. For example, *CpARF3*, *CpARF6*, *CpARF11*, *CpARF16*, and *CpARF17* showed the highest expression abundance (>50 %) in male flowers. However, *CpARF10* showed the highest expression abundance in hermaphrodite flowers, while it was almost undetectable in male flowers (Fig. 6 and Additional file 9: Table S6).

### Analysis of AuxREs in the promoter of reproduction-related genes

**Fig. 7:**
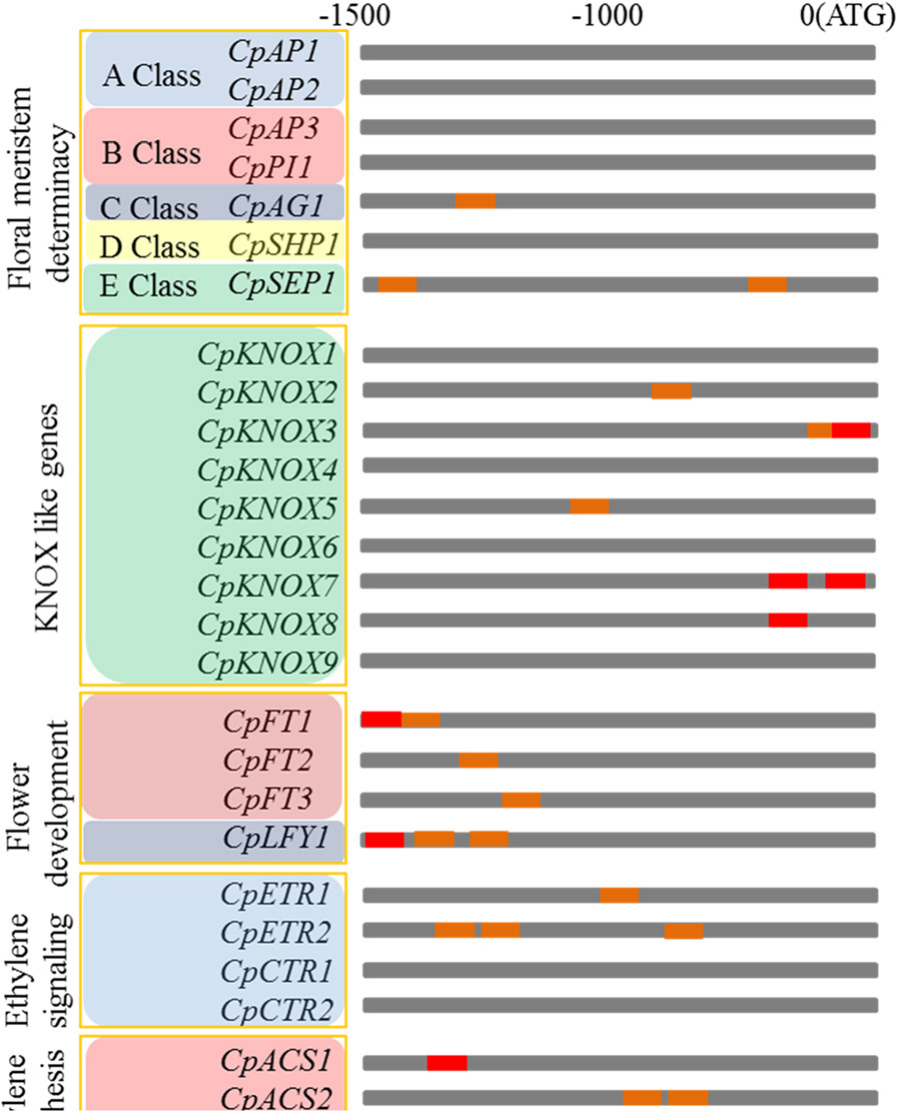
Analysis of *cis*-elements in flower and fruit development-related genes. The 1500 bp upstream from annotated start codons of 37 flower and fruit development-related genes were analyzed for the presence of AuxREs, which are given using the presented colour code. The red code indicated AUX1 (TGTGTC) and brown code indicated AUX2 (TGTXYS)

After searching the papaya genome database, we selected seven floral meristem determinacy related homologous genes (Class A–E) [43, 44], nine *CpKNOX* genes (*CpKNOX1*–*CpKNOX9*), four flower development-related homologous genes (*CpFT1-3* and *CpLFY1*) [45, 46], four ethylene-signaling-related homologous genes (*CpETR1/2* and *CpCTR1/2*) [47, 48], and three ethylene-synthesis-related homologous genes (*CpACS1/2* and *CpACO1*) [49] for this analysis. Among the 27 selected gene promoters, 16 promoters contained one or more AuxREs (AUX1 or 2) (Fig. 7 and Additional file 10: Table S7). Therefore, it was suggested that some reproduction-related genes could be strongly regulated by auxin treatment. All the promoter sequences of reproduction-related genes were listed in Additional file 11: Table S8.

### Endogenous IAA measurement

To reveal the involvement of auxin in the development of flowers and fruits in papaya, endogenous IAA contents were measured. The data showed that the endogenous IAA contents were much lower in the flowers under later stages than in the flowers under early stages. In the fruits, the endogenous IAA contents keep on a high level from stage one to stage four, and then significantly declined in the stages five and six. Furthermore, three different sex type flowers were collected for endogenous IAA measurements. The highest IAA contents were detected in the male flowers. The IAA contents in the female and hermaphrodite flowers were lower than that in the male flowers (Additional file 12: Figure S4).

## Discussion

Auxin is a key signaling molecule for most organogenesis and patterning processes occurring during plant development [50]. The auxin transduction pathway is mainly comprised of two transcriptional regulator families: ARFs and Aux/IAAs [37, 51]. ARFs directly bind to down-stream target genes and regulate their expression during development [52]. ARFs are also involved in the reproduction of various plant species [3, 53]. Characterization and analysis of CpARFs allowed us to reveal the mechanisms behind auxin involvement in fruit and flower development of papaya [54].

In this study, the reference genome sequence of papaya, which is relatively small in size (372 Mbp) [55], was used to identify the complete *CpARF* family. The number of CpARF genes was less than that in *Arabidopsis* (23 ARFs) [37]. Protein domain analysis provided us useful information on the biological function of ARFs. A typical ARF contains a DBD, an MR, and a CTD [37]. Aux/IAAs bind to CTDs of ARFs and form heterodimers. The presence of a large number of CpARFs without CTD suggested that some auxin-responsive genes in papaya can be regulated in an auxin independent manner [51]. The percentage of CTD-truncated CpARFs (36.4 %) was higher than that in other plant species, such as soybean (15.68 %), *Arabidopsis* (17.39 %), *Brassica rapa* (22.58 %), rice (24 %), and tomato (28.57 %) [10, 56]. Based on the amino acid composition of MR domains, CpARFs were classified into two groups: transcriptional activators and repressors [8]. The average activator/repressor ratio of CpARFs was 0.57 (Fig. 2), similar to *Arabidopsis* (0.59) and rice (0.56), and almost double compared to that in tomato (0.27) [5]. Only one ARF in papaya, CpARF6, contained only a DBD. These data provided insight into the potential functions of CpARF genes in plant developmental regulation.

We also built a phylogenetic tree to analyze the relationship of *ARF* families between papaya, *Arabidopsis*, and rice. The results showed that seven sister gene pairs with high bootstrap values (≥99 %) were identified between papaya and *Arabidopsis*, suggesting that *ARF*s in papaya were highly homologous to those in *Arabidopsis* (Fig. 1a). Many *AtARFs* have been already reported in previous reports [53, 57–59]; therefore, comparative studies may reveal useful information on the respective biological functions in papaya.

In *Arabidopsis*, transcription factors ARF6 and ARF8 regulate a complex process by promoting expansion, stamen filament elongation, anther dehiscence, and gynoecium maturation [18, 50]. The expression of *CpARF6*, a homologous gene of *AtARF6* and *AtARF8*, was increased more than six folds from flower developmental stage 1 to stage 8 (Fig. 4), indicating a putative function of this gene in flower development and maturation. The double mutant *arf6/arf8* in *Arabidopsis* delays the elongation of floral organs and subsequently delays the opening of flower buds and petal growth [18, 60]. Most defects in *arf6/arf8* are attributed to the abnormal expression of class one *KNOX*s [61]. The promoters of some KNOX genes in papaya, such as *CpKNOX2*, *3*, *5*, *7*, and *8*, contain several AuxRE elements, suggesting that these genes may be negatively regulated by *CpARF6* in the developing floral organs (Fig. 7).

*AtARF4* was also reported to be involved in flower patterning [62]. *CpARF4* (homologous gene of *AtARF4*) showed high expression levels in the flowers (Fig. 3). However, the expression of *CpARF4* gradually declined from flower developmental stage one to stage eight, suggesting that it might play a different role compared to *CpARF6* during flower development, especially at the initial stage. The *Arabidopsis* mutant *arf2* has a delayed flowering and ripening, while a double mutant *arf1/arf2* has an enhanced *arf2* phenotype, indicating that *AtARF1* acts in a partially redundant manner with *AtARF2* [53]. In papaya, *CpARF1* (homologous gene of *AtARF1*) was highly expressed in flowers, while *CpARF2* (homologous gene of *AtARF2*) showed a fruit-specific expression. Furthermore, the expression level of *CpARF1* was much higher in female flowers than in male flowers, and *CpARF2* showed an opposite expression pattern to *CpARF1*. The expression level of *CpARF2* was eight-fold higher in male flowers than in female flowers (Fig. 6). Additionally, the expression of *CpARF1* and *CpARF2* also declined during flower development (Fig. 4). The preferred expression in early stages suggested that *CpARF1* and *CpARF2* participated in flower bud formation, which is a key step for flower development. TIR1/AFB-mediated auxin-responsive gene expression is controlled by the interaction between Aux/IAA repressors and ARF transcription factors [63]. CpARF1, CpARF2, and CpARF4-related auxin expression regulation was decreased, while CpARF6-mediated auxin expression regulation was activated in the mature flowers.

Fruit development is a complex interplay of cell division, differentiation, and expansion that occurs in a temporally and spatially coordinated manner in the reproductive organs [64]. Auxin triggers and/or promotes the unpollinated, quiescent ovary to undergo cell division and elongation, and hence it is considered to play a major role in fruit set and development [65, 66]. In tomato, *SlARF*s are involved in the regulation of various aspects of fruit development [67]. SlARF7 acts as a negative regulator of fruit set after pollination and fertilization, and moderates auxin response during fruit growth [68]. Another tomato gene, *SlARF4*, an auxin response factor involved in the control of sugar metabolism during fruit development, expresses in pericarp tissues of immature fruit [26]. In papaya, several *CpARF* genes, including *CpARF2*, *CpARF6*, *CpARF7*, *CpARF10*, *CpARF16*, and *CpARF17*, displayed fruit-specific expression patterns, suggesting their importance in improving fruit-related agronomic traits in papaya [29]. Goetz *et al.* suggested that AtARF8 restricts auxin signal transduction in ovules and pistil until the initiation of fruit development [12]. However, no homologous gene of *AtARF8* was identified in papaya.

It is well studied that reproductive organs of plants reacted differently to different plant hormones. Many previous researches have presumed that auxin might play important roles in flower differentiation in papaya, and delay fruit ripening in other plant species [34, 36]. However, there is still no decisive evidence revealing that endogenous IAA plays roles in the flower and fruit development in papaya. The endogenous IAA contents showed a decline during both the flower and fruit development, suggesting that a high level of endogenous IAA might contribute to the initiation of reproductive organs in papaya.

Ethylene-auxin crosstalk regulates a variety of developmental and growth processes in plants, including fruit development and ripening [69–73]. Auxin plays a key role in progressing of fruit development towards the transition phase that leads to the initiation of autocatalytic ethylene production in an auxin- and ethylene-dependent manner [73–75]. In *Arabidopsis*, AtARF7 and AtARF19 are involved in ethylene response, indicating an interaction between auxin and ethylene [58]. *SlARF7*, a homolog of *AtARF7* in tomato, was also found to be involved in auxin signaling transduction during tomato fruit set and development [76]. In our study, the expression of *CpARF7* (homologous gene of *AtARF7*) was significantly inhibited during fruit ripening (Fig. 5). High expression levels of *CpARF6 and CpARF7* in mature flowers and early fruit developmental stages indicated that these two genes might be involved in fruit set and early cell division stage of the fruit. To get the putative targets for CpARFs during fruit ripening, we analyzed the promoter regions of several ethylene-signaling-and ethylene-synthesis-related genes in papaya [43–49]. The results showed that many AuxREs were contained in the promoters of two selected ethylene-signaling-related genes (*CpETR1* and *CpETR2*) and three ethylene-synthesis-related genes (*CpACS1*, *CpACS2* and *CpACO1*) (Fig. 7). In papaya, ARFs may be also involved in fruit ripening by regulating ethylene-signaling-related and ethylene-synthesis-related genes.

## Conclusions

In conclusion, our study provided comprehensive information on *ARF* family in papaya, including gene structures, chromosome locations, phylogenetic relationships, and expression patterns. The involvement of CpARF gene expressions in flower and fruit development allowed us to understand the role of ARF-mediated auxin signaling in the maturation of reproductive organs in papaya.

## Methods

### Plant materials and growth conditions

Two-year-old *C. papaya* cv. ‘Sunrise’ trees were planted in a 3 m × 3-m plot with drip irrigation at the Lingnan Normal University field experimental station in Zhanjiang City, Guangdong Province, China. Agronomic practices and fertilizer applications were applied as needed. Our experimental station has a gentle tropical oceanic monsoon climate with an average daily temperature of 22.8 °C, minimum temperature of 15.7 °C, and maximum temperature of 28.8 °C. The total yearly rainfall ranges between 1100 and 1800 mm [77]. The environmental conditions were strictly recorded during the sampling period. No extreme events and bad weather occurred in our experiment period.

### Genome-wide identification of *CpARF* genes

*Arabidopsis* ARFs (AtARFs) were used to blast against the *C. papaya* genome database on Phytozome 10.1 using TBLASTN (http://phytozome.jgi.doe.gov). Information on *AtARF*s used in this study is presented in Additional file 13: Table S9. Furthermore, the hidden Markov model (HMM) profiles of the ARF family [Pfam 02309: AUX/IAA family; Pfam 06507: ARF (AUX_RESP); Pfam 02362: DBD] were employed to identify *ARF*s from the *C. papaya* genome. All the obtained sequences were sorted as unique sequences for further protein domain search using InterProScan (http://www.ebi.ac.uk/Tools/pfa/iprscan/).

### Sequence analysis and phylogenetic tree

Multiple sequence alignment of CpARFs was performed using ClustalW (http://www.ebi.ac.uk/Tools/msa/clustalw2/) with the default parameters and adjusted manually. Four classical domains were identified in most CpARFs based on alignment results. DNA and cDNA sequences corresponding to each predicted gene were obtained from the *C. papaya* genome. *Arabidopsis* and rice *ARF*s (*OsARF*s) were used for the construction of a phylogenetic tree. Information on *AtARF*s and *OsARF*s is presented in Additional file 13: Table S9. Gene structure was analyzed using Gene Structure Display Server (http://gsds.cbi.pku.edu.cn/index.php), and the phylogenetic tree was constructed with 11 aligned CpARF sequences, 23 AtARF sequences, and 25 OsARF sequences using MEGA5.1 (http://www.megasoftware.net/) employing the neighbor-joining (NJ) method. Bootstrap values were calculated using 1000 iterations. The constructed phylogenetic tree was visualized using TreeView1.6 (http://www.brc.dcs.gla.ac.uk/services/).

### Prediction of amino-acid content and protein classification

Amino-acid content of the MR domain in CpARFs was calculated using MEGA 5.1, and the histogram was constructed using Excel 2010. The classification of CpARFs was based on the respective amino acid content [Domains with CTD: Glutamine/serine/leucine (QSL)-rich MR; Repressor with a carboxyl terminal domain (CTD); Serine/proline/glycine/leucine (SPGL)-rich MR; Repressor without CTD: Glycine-rich MR].

### RNA isolation and quantitative real time polymerase chain reaction (qRT-PCR)

Total RNA from different tissues, such as shoots, leaves, flowers, fruits, and roots, was extracted using Plant RNeasy Mini kit (Qiagen, Hilden, Germany) according to the manufacturer’s instructions. The criterion of flowers and fruits under different developmental stages was described as follows. In total, flowers of eight different developmental stages were collected in this experiment, including five stages of flower buds based on their diameters (1 mm, stage 1; 3 mm, stage 2; 5 mm, stage 3; 7 mm, stage 4 and 9–10 mm, stage 5), young flower with closed petals (stage 6), mature flower with partially opened petals (stage 7) and mature flower with opened petals (stage 8). In addition, papaya fruit samples of different developmental stages were harvested at 20, 40, 60, 80, 100 and 120 days after anthesis, respectively. For all the fruit samples, fruit core was excluded, and the flesh with peel were chopped up, frozen in liquid nitrogen and stored at−80 °C for further test. Flowers used in tissue-specific expression experiment were a mixture of male, female, and hermaphrodite types. To avoid the affects of environmental factors, the fruit and flower samples were collected from fifteen of uniform, well growth and disease free trees that distributed in different places in our field. Then, the samples were mixed and divided into several independent groups for further analysis.

DNase I was used to remove any genomic DNA contamination from total RNA. *CpActin* (evm.model.supercontig_18.238) was used as an internal standard to calculate the relative fold differences based on the comparative cycle threshold (2^-ΔΔ*Ct*^) values. Briefly, 1 μl of 1/20 dilution of cDNA was mixed with 5 μl of 2 × SYBGreen and 100 nM of each primer (forward and reverse), and then water was added to a final volume of 10 μl. PCR conditions were as follows: 95 °C for 10 min, 40 cycles at 95 °C for 15 s, and 60 °C for 60 s. All the primer sequences are listed in Additional file 14: Table S10. To visualize qRT-PCR data, heat map was constructed by ClustalW and Treeview using the average *Ct* value. In the heat map, red color represented up regulation, black color represented unchanged expression, and green color represented down regulation. In this experiment, a specific fold change value (2×) was used to identify any significant differences between different treatments. Expression analysis was carried out using five biological repeats, and the values shown in figures represent the average values of the five repeats.

### IAA treatment and *cis*-elements analysis

Flower and fruit samples were soaked in liquid Murashige and Skoog (MS) medium with or without (mock treatment) 10 μM IAA or 10 ìM 2, 3, 5-triodobenzoic acid (TIBA) for 1 h. Samples from each treatment were collected, and total RNA was isolated as previously described. Experiments were repeated five times with similar results. The promoters (1500 bp) of reproduction-related genes were obtained from Phytozome 10.1. AUX1 (TGTCTC core sequence) and a less stringent variant called AUX2 (TGTVYS) were used to manually scan promoter regions.

### IAA content measurement

The fruit and flower samples were collected and washed five times in deionized water to clean the surface of the tissues. The plant tissues were blotted dry with a paper towel and weighed using an electronic balance. After the addition of 500 pg of the ^13^C6-IAA internal standard, five independent biological replicates of each 50 mg sample were purified using ProElu C18 (http://www.dikma.com.cn). IAA contents were determined by a FOCUS GC-DSQII (Thermo Fisher Scientific Inc., Austin, TX, USA).

## Availability of supporting data

All the supporting data are included as Additional files.

## Additional files

Additional file 1: Table S1. The nucleic acid sequences of *CpARF* family genes. (DOCX 23 kb) Additional file 2: Figure S1. Multiple alignment profile of CpARF proteins obtained with ClustalW program. Multiple alignments of the DBD, MR and CTD domains of the CpARF proteins also were showed by different color lines. Colorized shading indicates identical and conversed amino acid residues, respectively. Two NLSs were marked by black asterisks. (TIF 3259 kb) Additional file 3: Table S2. Domain positions in 11 CpARF proteins. (DOCX 16 kb) Additional file 4: Table S3. Data of amino acid content in MR domain of CpARFs. (DOCX 19 kb) Additional file 5: Table S4. The saw data of the *CpARF* family during the different flower developmental stages. (XLSX 10 kb) Additional file 6: Table S5. The saw data of the *CpARF* family during the different fruit developmental stages. (XLSX 10 kb) Additional file 7: Figure S2. The expression level of *CpARF* genes under IAA and TIBA treatments in flowers. The histogram shows the relative expression level of *CpARF* genes under IAA and TIBA treatments during different time points compared to the mock expression level. Significant (P < 0.05) differences in control and treatments are indicated by an asterisk. (TIF 424 kb) Additional file 8: Figure S3. The expression level of *CpARF* genes under IAA and TIBA treatments fruits. The histogram shows the relative expression level of *CpARF* genes under IAA and TIBA treatments during different time points compared to the mock expression level. Significant (P < 0.05) differences in control and treatments are indicated by an asterisk. (TIF 407 kb) Additional file 9: Table S6. The saw data of the *CpARF* family during the different fruit developmental stages. (XLSX 10 kb) Additional file 10: Table S7. Promoter analysis (locations from ATG) of the genes involved in flower development and fruit ripening. (DOCX 16 kb) Additional file 11: Table S8. The promoter sequences of reproduction-related genes. (DOCX 28 kb) Additional file 12: Figure S4. The endogenous IAA contents measurment. (a) The endogenous IAA contents in the flowers under different developmental stages. (b) The endogenous IAA contents in the fruits under different developmental stages. (c) The endogenous IAA contents in the flowers of different sex types. Significant (P < 0.05) differences in IAA contents are indicated by an asterisk. (TIF 516 kb) Additional file 13: Table S9. The information of *ARF* family gene in *Arabidopsis* and rice. (DOCX 18 kb) Additional file 14: Table S10. Primer sequences for qRT-PCR of *CpARF* family genes. (DOCX 17 kb)

## Abbreviations

ARF: Auxin response factor
Aux/IAA: Auxin/indole-3-acetic acid
AuxRE: Auxin response element
CTD: C-terminal dimerization domain
DBD: DNA binding domain
GH3: Gretchen Hagen3
HMM: Hidden Markov model
kDa: k Dalton
SAUR: Small auxin up RNA
MR: Middle region
MS: Murashige and Skoog
ORF: Open reading frame
IAA: Indole-3-acetic acid
TIBA: 2, 3, 5-triodobenzoic acid
qRT-PCR: Quantitative real-time polymerase chain reaction
TBLAST: Basic local alignment search tool
