# Supplementary material for: Genome-wide identification and characterization of auxin response factor (ARF) family genes related to flower and fruit development in papaya (Carica papaya L.)
Source: BMC Genomics. 2015 Nov 5;16:901. doi: 10.1186/s12864-015-2182-0 (PMC4635992; doi:10.1186/s12864-015-2182-0)
Supplement: Additional file 1: Table S1. — The nucleic acid sequences of CpARF family genes. (DOCX 23 kb) [file 12864_2015_2182_MOESM1_ESM.docx]

**Table S1** The nucleic acid sequences of *CpARF* family genes.

>evm.TU.supercontig_9.161 (CpARF1)

ATGGGTCGTGATCAGAGGGAGGGGGTGAGGATTGCTCAGACCAGAAAAGCTGTGGATGTCCATGGCGGGAATCAAGGCGG

TCATGAAAGTGACCTATTCAATGAACTGTGGCATGCATGTGCTGGTCCTCTTGTTTACGTTCCCCGGGATGGAGAGAAGG

TTTTCTATTTCCCTCAAGGTCATATTGAACAGATTGAGGCATACACAAATCAAAGTGGTGAAGTGGAAATGCCCATCTAC

AATTTGCCCCCAAAGATTCTCTGCAAGGTTGTGTATGTTCGCTTAGAGGCTGAAATCACCACAGATGAGGTGTTTGCACA

TATTACTTTGCTTCCACTGAGAGAGCAAGATGGGTCAATTTTGGAGAATGGATGTTCCCTACCTGTGCCTCGTACATCTC

ACTCAAGTTTCTTTATCAAGAAACTTACTCCATCTGATACGAGCACCCATGGCGGATTCTCAGTCCCAAAACGTCATGCT

GAAGACTGCCTTCCACCTCTGGACTTGGCTCAGCAACCCCCAGTTCAGGACTTGGTTGCCACAGATTTGCATGGGGATCA

GTGGCACTTTCGCCATATATTTCGTGGTCAGCCGAAACGCCATTTGCTCACAAGTGGCTGGAGTACATTTGTTTCTTCAA

AGAAGCTTGTTGCTGGGGATGCATGCATCTTTCTCAGGGGACAAAATGGAGAACTTCGTGTTGGAGTTCGTTGTGGAGCA

AAACTACACAATAATGCAGCAGCAACCGTTCTATCTGCCCACAGCATGCAGCACGGAATACTTGCAAGTGCATACCATGC

AGTTTGCACTGGAAGTATGTTTAGTGTCTACTACCATCCTTGGACAAGCCCTGCCGAATTCATCATTCCCTATGATAAAT

ACATGAGGACAACTGAAATTGAATATTCTCTTGGAGCGAGATTCAAAATGATATTTGAAGCTGAAGAAGGTGGAGAGCAG

AGACTTGCAGGCACTGTAGTTGGTAATGAAGATATTGATCATATTAGGTGGCCTAACTCGGATTGGAGATGCCTTAAAGT

GAAGTGGGATATCAAATCAGATAGACTACCCTGCCCAGAGAGACTTTCTCCTTGGAGCATAGAGCTTATGGAATGCAACA

GGAAGAAGAAAACTTCTGTTCAACACCTGCAAAAGAGGGCTCGTGTTCATAGTTTATCAATTCCAGGAGTTTCCAGCATG

GTCCAAGATGGCTTGTTTCGAAATGCAGTTAAGCATTTTCCTCATTTAGGGGTCTTGCAAGGTCAAGAAAGTAAGGGCAA

GGGCATCATCATTGAAACAGATGCTCCAGGAATGCGACCCAGAAAGAATCCTGGTTGGAAACCAACACAACCAGAACTCG

AGGATCATCTGGCCTTTGCAATGGATGATCCATTTGATAAATGTCACATTAGCTCAATTCTATTCTCTGGTGGAAATATT

GGAAGTTCAATTCCAAAGGGTTGCCCTTCAGCCCATGCTGTGCAGAACAAAGCTAAAGGTAAACAAATCTTATCAGTTCC

AAATGTCAACTTCTCCAATTCTGAATCTTCAGACCGGAGGGCTTTGGAACTGAGGGACCAAAATGATGCTCCCCCAATGC

AACAAAGTTGCCTGGGTGGATACAAACTTTTTGGAGTCAATTTGTTCAATAGTCCACCAGAGCTCCCTTCACCCCAAGTT

GCCAATTCCAGTGATTTTGAATGTTTTTCTTATGTTCCACCAACATCCCAGTCAAGTATTTCTGGAACTAAAAGGGTCAC

AGACCCATCAAACAGCCTATCTGGCAGCCTCTCAAAAAAGAAATGCAACACTTTCTGCTCCGTCACTAATCGGAGTTGCA

CCAAGGTCCTCAAGTATGGAACTGCCCTTGGAAGATCAATTGACCTCACAAAATTTAATGGGTATGAAGAACTTGTTTGC

ACGCTTGACCAGATGTTTGACTTCAAAGGAAGCTTGATGGATGGGACTAGTGGTTGGCATGTAGCCTACACAGATGATGA

AGGAGACATGATGCTGATGGGTGATTATCCATGGCAGGGATTCCAGGGCATGGTGAGGAAGATGTTCATCTGTCCTAAGG

AAGATCCAGAAAACTGA

>evm.TU.contig_31756.1(CpARF2)

ATGGGTTCTTCAGAGGTGTCAATAAAGGAAAATTCCGTTCACGGGAGAGGAGAGAGCTTTTCTTCGGGTTACAGTGAGCC

TAACCATGGTAGGAACACTATGGAGGGGCAGAACGGTCATTCTACGACTTCCAATGCTTCTAGAGTGGACCCAGAAGCGG

CGCTTTACACGGAGCTATGGCATGCTTGTGCCGGTCCTCTGGTTACTGTCCCTCGAGAAAGAGAGCGTGTCTTTTACTTC

CCTCAGGGACACATCGAGCAGGTCGAGGCGTCTACCAATCAGGTTGCAGACCAAAAGATGCCAGTCTATAATCTTCCTTC

GAAGATCCTTTGTCGTGTGATTAATGTCCAATTGAAGGCTGAACCGGATACTGATGAGGTGTTTGCGCAAGTTACTCTAC

TTCCAGAGCCGAACCAAGATGAGAATGCGGTGGATAAGGAGCCATCCCCACCTCAGCCGCCGCGGTTTCATGTACATTCG

TTTTGTAAGACGCTGACTGCTTCCGACACAAGTACTCACGGTGGATTTTCTGTTCTTAGGCGACATGCCGATGAGTGTCT

GCCGCCTCTGGATATGTCACGGCAGCCTCCCACCCAGGAGCTGGCGGCAAAGGATTTACATGGAAATGTGTGGCACTTCA

GGCATATTTTCCGAGGTCAGCCGCGCAGGCACTTGCTTCAGAGCGGTTGGAGTGTGTTCGTTAGCTCCAAGAGGCTTGTT

GCAGGAGATGCATTTATATTCTTAAGGGGTGAGAATGGGGAACTTCGTGTTGGTGTAAGGCGTGCAATGAGACAGCAAGG

CAATGTTCCGTCCTCCGTAATATCTAGTCATAGTATGCATCTTGGCGTTCTTGCTACGGCATGGCATGCAATCTCGACGG

GAACTATGTTCACGGTTTACTATAAGCCTAGAACTAGTCCTGCCGAGTTTATTGTTCCATATGATCAATATATGGAGTCC

GTTAAGAACAGCTATGCAATCGGGATGAGATTCAAAATGAGATTTGAAGGTGAAGAAGCTCCAGAACAGAGGTTTACTGG

CACTATTGTTGGAATTGAAGATGCTGACCCCCAAAGATGGCGAGAATCCAAATGGAGATGCCTTAAGGTAAGGTGGGATG

AAACATCAACTATACCTCGACCAGAAAGAGTTTCCCCTTGGAAAATAGAGCCTGCCCTGGCTCCTCCGGCATTGAATCCC

CTTCCCATGCCCAGGTCCAAAAGGCCTAGACCAAACATGCTGCCTTCATCACCTGACTCATCTGTGCTCACTCGGGAAGG

TTCATCTAAGGTAACTGTAGACCCTTCGCCAGCAAGTGGTTATTCGAGGGTCTTGCAAGGTCAAGAATTCTCGACCTTGA

AGGGCAATTTTGCAGAGAGTAATGAATCTGATACTGCTGAGAAGTCTGTTGTATGGCAACCTTCAATAGATGATGACAAG

GTTGACGTAGTTTCTGCTTCAAGACGATATGGTTCAGAGAGTTGGATGCCTACAGGAAGGCATGAACCAACTTATACAGA

TTTACTATCTGGATTTGGAGCCAATGCTGACCTCACCCACGGTCACTGCTCATCATTCGCTGATCAAGCTGTGGCAACTA

TGAATCCAATTAAGAAACATTTACTTGATCAGGAGGGAAAGTTTAACTTGCTTCCAAGTCCGTGGTCCCTAATGTCTTCT

GGTCTCTCTCTTAAGTTGGCTGAATCAAGTTCTAATGTTCCCAAGCAAGGTGTTGATGCATCTTACCCATCACGAGGAAA

CATTAGACATGGAGGATATGGTGAATATCCCATGCTTCATGGTCACAGAGTGGAGCATTCCCATGGAAGCTGGATGATGC

CACCTCCACCACCGTCTC

> evm.model.supercontig_7.3 (CpARF3)

ATGGGGGGCTTGATCGATCTCAACACTACCGAGGAAGATGAAACGCCGTCCTCCGCTTCGCTCTCACCATCTTCCTCCTC

TGCTTCTGTTCTCAGCGCTTCTGCTGTAACCGCTGCTGCTTCTGCTGTGTGTCTGGAGCTCTGGCACGCATGTGCAGGGC

CACTGATATCTCTGCCTAAGAGAGGGAGTATTGTGGTGTACTTTCCTCAGGGACACTTAGAACATGTTCCCAATTTCCCC

GCCGCAGCTTATGATCTTCCTCCCCATATTTTCTGTCGTGTTGTTGATGTCAAGCTCCATGCAGAGGCAGCAACCGATGA

GGTCTATGCGCAGGTCTCGCTTGTTCCCGAAACTGAGCAAATTGAGCAAAAGTTACATGATGGGTCTATTGAGGCTGCCG

ATGGTGAGGAGGATATAGAAGCAGCTAGAAAGTCTATCACACCTCACATGTTCTGCAAGACCCTTACTGCCTCAGATACT

AGCACCCATGGAGGCTTCTCTGTCCCCCGCCGGGCTGCCGAGGATTGCTTCCCTCCTCTGGATTACGATCAGCAAAGACC

TTCACAAGAGCTTGTAGCAAAGGATCTTCATGGAGTGGAATGGAGATTCCGACACATCTATAGGGGCCAGCCTCGAAGGC

ATTTGCTTACGACTGGATGGACTGCTTTTGTCAATAAAAAGAAGCTTGTTTCTGGAGATGCTGTGCTCTTTCTTCGGGGT

GAAGATGGAGAATTGAGACTTGGAATCCGAAGAGCAGCTCAAGTTAAAGTTGGTTCTACTTTTTCTGCTCTCTGTGGCCA

GCAACTGAATCACAGCAATTATGCAGATGTAGTTCATGCTGTATCTATGCGAAGTGCTTTCAGTATTTACTACAATCCAA

GGGGAAGCTTGTCAGAGTTTGTAATACCTGTTCGCAAATTCCTAAAGAGCATTGATCACTCTTTCTCTGTTGGAATGAGG

TTCAAAATGCGTTTTGAGACAGAAGATGCTGCTGAGCGAAGATACACAGGACTGATAACTGGGATTAATGAGATGGATCC

TCTAAGATGGCCTGGTTCAAAATGGCGATGCCTCGTGGTAAGGTGGGATGATATTGAGGCCAACAGGCATCATAGGGTTT

CTCCGTGGGAAATTGAGTCATCAGGTCCCATGTCCAGTTCCTGTGGCTCAATAGCAACTGGTTCGAAGCGAACCAGGATT

GGATTAGCATCAGGAAAACCAGAGTTTTCAGTTCCTGATGGAATTGGAGCAACAGACTTTGGGGAATCTCTAAGGTTCCA

GAAGGTCTTGCAAGGTCAAGAAATTTTTGGTTTTAGTTCTCCCTGTAATGGTAATGATGGGAAGAATCTTCATCCTTCTG

AAATTAGAAGGTGCTTTCCTGGTTCGAATGGATGTGGGGTTGCTTCCATAGCACATGGCATTGGTGACCCACTAGCAAAT

TCTGATACTCCCTATCAAGGCACAGGCTTTGGTGAATCTTTTCGATTCCGTAAGGTCTTGCAAGGTCAAGAAATGCTTCC

AAGCACTCCATATGGAAGAACCGTGACTTCTTATGAGGGTCGTGAAAATGGTTGCTTTGGAAGCTCAGATGCTATCCAGG

TGCCTGGATCAAGAAATGGATGGTCCAGTCGAGTGCAGGGATATGGTTCTCGGATGCACCCTTCTGTCCAATGCATGCAA

GTGTCATCACCATCTTCTGTATTAATGTTCCAGCAAGCAGGGAATCCAGTTCCAAATGTCAATCCACTTTATGGTGTTAC

TGATCAAGAGGAGCAGAGGCAAACCGATCAATCTTCTATTCATACTCATGAAACATACAGGGGAAAGCTCAAATCATCAT

CAGTCCCAGGGTGCAGTTTGAGGGGGGAAGATGAGGGAGGCATCACTTCCTTTGTACCCCCTGGGTCGCCCGCTTCACCC

AGGTCGGCTCCCTCAGCTCTCACACCTGCGGTGCCACCATCCAGCTCCACAGGAAAGCTCATCCGCAGAGCTCTTGTCGC

CGTGTATGAATATGCTAAAGCTTAA

> evm.model.supercontig_139.80 CDS

ATGGAAATTGATCTGAACTATGCAGCGACAGAGGTGGTGGAGAAGAGTAATACAAATGTTCCTTGTAATGGGGACTGTCA

CAAGGCCAGTGGGTGTGTTAGCTGCTGTTTGACTTCTTCATCATCTTCTTCTTCCTCTTCCAATTCATCTTCAGCTCCTG

GTTCTTCTTCACCTTCTTCAATTTACTTGGAGCTTTGGCATGCCTGCGCTGGTCCTCTTACTTCGCTGCCCAAGAAAGGG

AATGTGGTTGTTTACGTCCCTCAAGGCCATTTAGAACAGGCTGCTGCTGCTGCCTCTGCATCTCCTTTCTCCACCATGGA

AATGCCCACCTTTGATCTCAATCCCCAAATCTTTTGCAGGGTTGTTAATGTCCAACTCCTTGCCAATAAGGAGAACGATG

AGGTATACACGCAGGTTACTTTGCTTCCTCAACCAGAGTTTGTAGGGCTGAATTCAGAGGGCAAAGAGGCTGCAGAATTG

GGACTGGAAGAAGAAGGAAGAGGAGGGTCACTTACAAAATCAACCCCTCACATGTTCTGCAAAACACTAACAGCTTCTGA

TACTAGCACTCATGGTGGATTCTCTGTCCCTCGTAGAGCTGCTGAAGACTGTTTCCCTCCTCTGGACTATAAGCAGCAGA

GACCCTCTCAAGAGCTTGTGGCCAAGGACCTTCATGGAGTAGAGTGGAGATTTAGGCATATATATAGAGGTCAACCAAGG

AGGCACCTGCTCACTACTGGATGGAGTATTTTTGTAAGTCAAAAGAATCTTGCTTCGGGCGATGCTGTGCTCTTTTTGAG

GGGCGAAGATGGAGAGCTAAGGTTGGGAATTAGAAGAGCTGCTCGACCAAGAAATGGACTTCCTGATGCAATTATTGCAA

AGCAGAATTCTTATCCCAGTATTCTTTCTCTCGTGGCTAATGCAGTATCCACCAAGAGCATGTTTCATGTTTTCTACAGT

CCTCGGGCTAGTCATGCGGAATTTGTTATACCCTACCAAAAGTATATCAAAAGCATCACCAGTCCAATATGTATTGGGAC

AAGATTCAGAATGAAATTTGAGATGGAAGATTCGCCCGAAAGAAGATGCAGTGGCGTAGTGATTGGAATTGGTGACTTGG

ATCCACATAGATGGCCTAACTCGAAATGGAGGTGTTTGATGGTTAGATGGGATGAAGACATGGTGAGTGATCACCATGAA

CGAGTTTCACCATGGGAAATTGATCCTTCTGTTTCTCTTCAACCCTTGAGTATTCAGTCTTCTCCAAGGCTGAAGAAACT

GCGTACAGGAATGCAGGCAACTCCACCTGACAATCCTGTAACCGCAGGAGGAGGTGGTTTTTTGGACTTTGAGGAGTCTG

TGAGACCCTCTAAGGTCTTGCAAGGTCAAGAAAATGTAGGTTTCGTATCACCCTTGTACGGATGTGATACTGTGAACCGC

TCGCTGGATTTTGATCTGCAATCCAATGCACATCAAAGTCTTGCGTCAACCAGAACAGAAAATTCTAAAATCAGTGAATT

TATGAGGGCTCAGCCCCAAACTTACGCAAGCTTTGCAGAATCCAATAGGTTTCCTGGGGTCTTGCAAGGTCAAGAAATTT

GTCCATTTAGGTCTCTGAGACAAAATGCTGACTACAATATTGGTTCATGGGGAAAGTCAAATCTCAATTTCAACTCTTTC

AATATCTATCAAGCAACCAAACCCAGTCTCTATCCGCTAGCTTCAGAAGGCCCTCGAAATATATACTTTCCTTATGGTGG

CATATATAGAGCCGGCCAAGATCCTATGATCCGACCTTATGCAACTAACTTCCCAAGAGAGAGTGTTACATTTGCAACAT

CTTCAATTCAGAATCGGATAATTGGGGATGGAATTAGAAACCCAAGTCTTCTGAAAGAGCATAAGCCACCGGAAAATATC

CCTACTGCATCTTCTTTCAAGACAAATATCGGATGTCAAAAGGAAGAAGGCTTTAATGGGACTGGATGTAAACTTTTCGG

ATTCTCCCTAACTGGAGAAACAGCAACTTCGAACCCTCAAAGCTCTGGCAAAAGGAGTTGTACAAAGGTTAGCAAGCAAG

GTAGCTTGGTTCGAAGGGCTATCGATCTCTCAAGATTAAACGGGTATGATGAACTGTTGAATGAACTTGAGCGCCTGTTT

AGCATGGAAGGGCTTTTACGAGATCCTGATAAAGGATGGAGGGTCTTGTACACTGACAGTGAGAACGATGTGATGGTCGT

TGGCGATGATCCATGGCATGAATTCTGTGATGTGGTGTCCAAGATCCACATATACACCAAAGAAGAAGTGGAGAAGATGA

CTCTGGGCGTGATCAATGATGACACGCAGAGTTGTCTGGAGCAAGCACCGGTCATAATGGAAGCATCAAAATCTTCCTCA

GTGGGTCAACCAGACTCTTCCCCAACCGTAATTAGGGTTTGA

> evm.model.supercontig_26.24 (CpARF5)

ATGGCTTCCGTTGAAGACAATATCAAAACAAGTGTTCTACTTACTGGGCCTGCACAAACTACTCTGTTCGAGGAGATGAA

ATTGCTGAAAGAAATGCAGGATCCATCTGGGGATCGAAAGGCTATAAATTCGGAACTATGGCATGCGTGTGCAGGGCCCC

TTGTTTTCTTGCCTCAAGTGGGGAGTCTTGTCTACTACTTTCCTCAAGGACATAGCGAACAGGTGGCTGTTTCCACTAAA

AGAACAGCTACTTCCCAAATCCCCAATTACCCAAATCTTCAGTCTCAGTTGATGTGCCAAGTTCACAATGTTACTCTACA

TGCAGACAAAGATACAGATGAAATTTATGCTCAAATGAGTCTTCAACCTGTGAACTCGGAAAGGGATGTTTTCCCTATAC

CAGATTTTGGACTTAGACTCAGCAAGCATCCGAGTGAGTTTTTCTGTAAAACTCTTACAGCAAGTGATACAAGCACACAT

GGTGGTTTCTCAGTGCCGCGTAGAGCAGCAGAAAAGCTCTTTCCTCCATTGGATTATACAATGCAGCCCCCAACCCAAGA

GCTTGTTGTCCGAGATTTGCATGATAATACTTGGACTTTTCGTCATATATACCGCGGGCAGCCAAAGCGGCACCTTCTCA

CAACAGGATGGAGTATGTTTGTTGGCTCAAAAAGGCTCAGAGCAGGTGATTCTGTTCTGTTTATCAGGGATGAAAAGTCA

CAGTTAATGGTAGGAGTGAGGCGTGCTAATCGTCAACAGACAACACTACCATCATCAGTTCTTTCGGCTGATAGCATGCA

CATAGGTGTCCTTGCTGCTGCAGCTCATGCTGCTGCTAATCGAAGCCCATTTACAATTTTCTACAATCCAAGGGCATGCC

CTTCAGAATTTGTCATTCCAATGGCAAAATACCGGAAATCTGTTTTTGGGACTCAACTCTCAGTTGGTATGAGGTTTGGG

ATGATGTTTGAAACAGAGGAGTCTGGTAAGCGCAGATATATGGGCACAATAGTTGGCATTAGTGACTTAGATCCTCTGAG

GTGGCCTGGTTCGAAGTGGCGTAATCTGCAGGTTGAGTGGGATGAGCCTGGATGCAATGATAAACAAAGCAGGGTTAGTG

CATGGGAAATTGAGACTCCTGAAAGTCTCTTCATATTTCCGTCGCTAACTTCAGGTCTCAAGCGCTCGTTACATCCTGGG

CTTTTGGCAACTGAAACTGAATGGGGAAACTTGATAAAAAGACCTATTATCAGGGTTCCTGACATTAGCAATGGGGATTT

TCCATACTCAGTTTCAAACTTATGTTCTGAAAAACTAATGAAGATGATGCTGAAGCCTCAGCTTGTTAACCATCCTGGAT

CTGGAGCCTTTGCATCCACCCTACAAGTCCCTGTTGCTACCGGAACTTCATTAGAAGGGGCGACAAACATGCAGGCTGCA

ATCAATCAGAAATCTCAAATTTTCCAGACAGAAAATAGATTAATAGAAAAGCAGAACTATCCCCAGTTCTGCCCAAGCCA

ACCGACTGCTGTTAGTTCAAATTCATCAAAAGTAACTGTTCCTGTTGATGCACATCCTGAAGTCAAAGCGGAAAGTTCAA

CACCAACTGGAAGTAATTCTGAAAAATTGAAGCTAGAACCTGAGCATTCAACTGATCAGTTAAGCCAATTAACTTCCACG

ACTGAGTGCAACAAGGAAAAGTTTGTCTCAACTTCTATGAATTCAGAGAGTCAGATGAACCAACCAACATTAAATAACCA

GATACATTTGCAATCTAATCCATGGACCATGCAGCCTCAGTTGGAATGCTCCCAACAAATGTACCCAGCTCAATCTGATT

CTACCATAACTGGGTTACCTTCATTCCCAGAATCTGATGAATGGACATCGCATCTTTCTTCTTGCCAGTCTCTTGCCACA

ACATATACATCACCTGGGCCTGTAACCATGTTTGGCACACAAGACTCTTCAGCAATGTTATCCGAAGTATTCAACCCTTC

AATGGATCAAGATATTTGGGATCATCAGCTGAATAATCTAAAGATCTTCTCACAGCCTGACCAGTTTACTTCCTTCATGC

AGCCTGATCCATGCAGCCTTAATTCAACTGGACTTAGAGATCTTTCAGATGAAAGCAATAATCAGAGCGGAATTTATAGC

TGTCTTAACATTGAGGCTGGTGGTGGTGGAAGTACTGTGATTAATCCTTCTGTTTCAAATGCCATCCTGGATGAGTTTTG

TTCATTAAAGGAAGCCGATTTCCAGAATCCAACCGATTGTTTAATAGGGAACTTTAGTACTAGTCAAGATGTTCAATCTC

AGATTACATCTGCAAGTCTTGCAGAATCCCAAGCATTGTCTCGGCAGGACTTCCCGGACAACTCAGGGGGTACATCTTCA

AGCAATATTGACTTTGATGAGAGCAGTCTATTGCAGAATAGTTCATGGCAGCAAGTAGCACCACGTGTGCGAACATATAC

AAAGGTTCAAAAAGCTGGATCTGTTGGGAGGTCAATTGATGTCACAAGTTTTAAGAACTATGATGAACTGTGTTCTGCAA

TTGAATGCATGTTTGGACTTGAAGGGCTGCTTAACGATCGGAGAGGTTCAGGATGGAAGTTGGTATATGTGGACTACGAG

AATGATGTTCTACTTGTTGGGGATGATCCCTGGGAGGAATTTGTTGGATGTGTTCGTTGCATCAGAATCCTGTCACCAAC

TGAAGTTCAGCAGATGAGTGAAGAGGGAATGAAGCTTTTGAACAGCGCTGCAATGCAAGGCATGAAAGTTGCTATATCAG

AAGGTGGCTGTGCTTGA

> evm.model.supercontig_17.53 (CpARF6)

ATGAGGCTCTCTTCAGCTGGTTTCAATCCACAAGCTCAAGAAGGGGAAAAACGGGTACTGAACTCTGAACTTTGGCATGC

ATGTGCTGGACCTCTTGTTTCTCTTCCTCCCGTTGGCAGCCGAGTTGTCTATTTCCCACAGGGTCATAGTGAGCAGGTTG

CTGCATCTACCAATAAGGAAGTGGATACTCATATTCCTAACTACCCAAGCTTACCTCCCCAACTTATCTGTCAACTTCAC

AATGTTACCATGCATGCAGATTTGGAAACTGATGAAGTCTACGCGCAAATGACTCTGCAACCACTGAGTCCGCAAGAGCA

AAAGGATGCCTACCTTCCTGCTGAATTGGGCACTCCCAGCAGACAGCCAACAAATTATTTTTGTAAAACGTTGACAGCTA

GCGACACGAGTACTCATGGGGGGTTCTCGGTCCCCCGCCGGGCAGCTGAAAAAGTGTTCCCACCATTGGACTACTCACAG

CAGCCTCCAGCTCAAGAGCTTATTGCGAGGGATCTGCATGATAATGAATGGAAATTTAGACATATTTTTCGGGGGCAACC

CAAAAGGCACCTCCTTACAACAGGATGGAGTGTGTTTGTAAGTGCTAAAAGGCTTGTTGCTGGTGATTCAGTCCTCTTTA

TCTGGAATGAAAAGAATCAATTACTTCTTGGTATTAGACGGGCTAATCGACCACAGACAGTAATGCCTTCATCAGTTTTA

TCAAGCGATAGCATGCACTTGGGGCTTCTTGCTGCTGCGGCTCATGCGGCTGCGACAAATAGCCGTTTTACAATATTCTA

TAATCCAAGGGCCAGTCCATCAGAGTTTGTTATACCACTGACCAAGTATGTCAAGGCGGTCTATCACACACGTGTTTCTG

TTGGGATGCGCTTCCGGATGCTGTTTGAGACAGAGGAATCAAGTGTCCGCCGGTAG

> evm.model.supercontig_261.2 (cpARF7)

ATGAAGGCTCCATCTAATGGGTTTTTGCCAAATTCTGGAGAAGGAGAGAGAAAAAGTATTAACTCAGAGTTATGGCACGC

ATGTGCTGGACCTCTGGTTTCTTTACCCCCCGTGGGAAGTCTGGTGGTTTACTTCCCTCAAGGCCATAGTGAGCAAGTTG

CAGCATCAATGCAAAAAGAGACTGATTTCATACCCAGCTACCCTAATCTTCCTTCCAAATTGATTTGCATGCTTCATAAT

GTGACATTGCATGCTGATCCTGAGACAGACGAGGTCTATGCACAGATGACCCTTCAACCTGTAAACAAATATGATAAGGA

TGCTCTGTTAACTTCTGATATGGGGCTGAAGCAAAGTCAACCCAAAAGGCACCTGCTGACAACTGGATGGAGCGTGTTTG

TTAGCACAAAAAGACTCTTCGCTGGAGATTCTGTGCTTTTTATAAGAGATGAAAAGTCACAGCTTCTTTTGGGTATAAGA

CGTGCTAATAGACAGCAGCCGGCTCTGTCTTCATCAGTCATATCCAGTGACAGCATGCACATAGGAATTCTTGCAGCAGC

AGCACATGCTGCTGCCAATTTCAGCCCATTTACTATATTCTACAATCCAAGGGCTAGCCCTTCTGAGTTTGTCGTTCCCT

TAGCCAAGTATAATAAAGCAATGTACACCCAAGTCTCTATTGGCATGAGGTTCAGGATGATGTTTGAGACTGAGGAATCG

GGGGTTCGTAGATATATGGGAACAATTACTAATATTAGTGATTTGGATCCAATTCGATGGAAAAATTCACAATGGCGCAA

TCTTCAGGTTGGATGGGATGAATCGACAGCTGGTGAACGGCCAAGTCGAGTTTCCATTTGGGAAGTTGAACCTGTCGTAA

CTCCTTTCTACATATGTCCCCCTCCGTTTTTCAGACCTAAATTTCCCAGACAACCAGGGATGCCAGATGATGATTCTGAT

ATAGAGAATGCTTTCAAAAGGGCAATGCCCTGGGATGATTTTGGGATTAAGGACGCCACAAGTTCAATCTTTCCTGGTTT

GAGTTTAGTTCAGTGGATGAGTATGCAACAGAATAATCAACTTTCAGCTGCACAATCTGGATTCTTTCCGTCTATGGTTT

CTTCAACTGCGATGCATAATAACATTGGTGTTGATGATCCATCTAAGCTATTGAATTTTCAAGCCCCAGCACTATCTGCC

CCAAATCTCCTTTTTAACAAAGCAAATCCACTGAATCAAGTTAACCAATTACCGCAGCCACCTACTACATGGCCCCAACA

ACAGCAACTGCAGCAGCTCTTGCAGACTCCTAGAGCTCAGCAGCCACAAGTTCAACAATTATCACAGCAAAGCCTCCCAG

AGCAACAGCTTCAACTGCAGCTATTACAGAAATTTCAGCAACAACAACAGCAACAGTTGCTCTCTCCAGCGAGCTCACTC

TTGCAGCCTCAGTTGCTGCAGCAACAGCAGCCTAGTTCACAAAATCAGCAAATGCTGCTGCCTCTGACCCAGAATCAACA

ACTACCGCTCAGCAGTAACAGCTTTTCAACATCAACGCTCATGCAGCCTCCACAACTTCCTCTTAACCAGCCTCAGGGCC

AGAATAAACCACTTAATGCTATCAGAGCCCACTCTGGTCTTACTGATGGAGAGGCTCCTTCATGTTCAACCTCACCTTCA

ACCAATAATTGCCAGGTTTCCCCACCAAACTTCTTGAACAGGAACCAACAAGGGCCAGCCATGTTGGCATCTGATTCAGT

GGTTGAGCCTCCAAGTAATCTATTCCAAGAGCTTCAGAGTAAGTCAGATATGCATATGAAGCATGAGTTGCCTGGTTCAA

AAAGTTCAGATCCACAGAAGTGCAAAGGTACTGTGACGGATCAGTTGGAGGCCGCCTCTTCCGGAACATCATATTGCCTG

GATGCTGGCACCATCCAACAAAATTTCTCTCTTCCCACATTATGTTTGGATGTTGATATCCAATCACATCCGCGGAACAA

CCTGCATTTTGCAGCTAATGTTGATAGCTTGACACCTGACACCCTGTTGTCAAGAGGGTATGACTCTCAAAAGGATCTTC

AGAACTTACTTTCTAATTATGGTGGCACTCCAAGAGATATTGAGACGGAGTTATCAACTGCTGCCATAAGTTCTCAGTCA

TTTGGTGTGCCAAGCATGCCTTTCAAACCCGGATGTTCAAATGATGTTGCCATCAATGAAGCTGGGGTTTTGAACGGTGG

ACTGTGGGCCAACCAAACTCAGCGAATCCGAACATATACAAAGGTTCAAAAGCGTGGCTCTGTGGGAAGATCGATTGATG

TCACCCGTTACAAGGGGTATGATGAGCTAAGGCATGATCTTGCACGCATGTTTGGGATTGAAGGACAGTTGGAAGATCCA

CAAAGCTCTGACTGGAAACTAGTTTATGTCGATCATGAAAATGACATACTACTTGTTGGTGATGATCCTTGGGAGGAGTT

TGTGAGCTGTGTCCAGAGCATAAAGATACTCTCATCTGCTGAAGTACAGCAGATGAGCTTGGATGGAGATCTTGGAAATG

TGCCAGTTCCTAATCAAGCATGTAGTGGGACAGATAGTGGGAATGCATGGAGAGGACACTATGATGACAACTCAGCCGCA

TCATTTAACTGA

> evm.model.supercontig_65.4 (CpARF10)

ATGAAGGAACAAGAGAAAAGCTTGGATCCTCAACTATGGCATGCATGTGCAGGATCCATGGTTCAAATCCCTGCTGTAAA

CTCCATCGTCTTCTACTTCCCACAAGGCCATGCCGAGCACGCTCAGGCCACCGCCGTCGATTTCTCATCCTCGCCGCGAA

TTCCTTCCTCCGTTCTATGCCGGGTGGCCGCCGTCAAATTCCTCGCCGACACCGAGACAGACGAGGTTTACGCCAAGATC

AGACTGATCCCACTACCGAACAGCGAGGTTGATTTCGACGACAACGTGGTTTTCGGGTCGGATAATCCGGAGAAACCGGC

TTCTTTCGCCAAGACCCTAACTCAATCCGACGCCAATAATGGCGGGGGATTCTCCGTTCCCCGGTACTGCGCGGAAACAA

TATTCCCCCGGCTGGATTACACGGCGGATCCTCCGGTTCAGACGGTGATCGCAAAGGACATTCATGGAGAAACATGGAAG

TTTAGACACATCTACAGAGGGACTCCGAGGAGGCATCTGTTGACCACCGGGTGGAGCACTTTCGTTAACCAGAAGAAACT

CGTCGCCGGCGACTCCATCGTGTTTTTACGAGCGGAAAACGGTGATCTCTGCGTGGGAATCCGGAGAGCCAAACGTGGGG

TTGGTAGTGGACCGGAGATGATTTCCCCGTCTTCTTCTTCCTCTGGCTGGAGCACCGGTCAGGGAAACTGTACCCCATTT

GGTGGGTTTTCGGCGTTTTTGAGAGGAAATGAAGAGAGCAAGATGTTAAGAAATGGAAATGGAAGCTTTAGAGGGAAACT

GAGGCCGGAATCGGTAGTGGAAGCGGTTGCTCTGGCGGCTGCAGGGCAGCCATTTGAGGTAGTTTATTATCCGAGAGCAA

GCACGCCGGAGTTTTTTGTGAAAGCATCGGCTGTTAAAGCCGCCATGAGAATCCATTGGTGCTCTGGTATGAGGTTCAAG

ATGGCTTTTGAGACTGAGGATTCTTCCAGGATCAGCTGGTTCATGGGGACTGTTTCCTTTGTTCAGGTTGCTGACCCTAT

CCGCTTTCCCAACTCGCCATGGCGCCTTCTCCAGGTGGCATGGGATGAGCCGGATTTACTACAGAATGTGAAGCGTGTGA

GTCCATGGCTGGTGGAATTGGCAACCCCCCTTGGGCCTGGCAGCCCGTTGTGTTATCTATCCGATAACACTCCTGCAGGC

ATACAGGGAGCCAGGCAAGCTCAATTTGGAATATCTTTATCAGATCTCCATCTTAATAACAAGCTACAATCAGGGCTGTT

TCTTTCTAGCTTCCAGCGGTTAGATCAACACTCTAGAACTTATGATAGTGTCAGAACAACAGGGCACACGGAAGGCAACA

ATGAGAATTTGTCTTGCTTGTTAACGATAGGTAATTCTAGTTGTAATTCGGAGAAATCTGATAAGACGAAGAGACACCAG

TTCGTACTCTTTGGTCAGCCAATACTCACAGAGCAGCAGGTTTCTATTGATACAACAGTCACTTCGAGAAGTTTATTAGA

AGAGAGCAAGGAAAAAATTAAAGAGCTTTCTTTGCAATCTACTTCTAGGGATCGAGTTTCCCCCGAAAGGTTTGCACCCG

CTGGATTCTCATGGCACCAGAGCTTTCAAACCACCGAATCTGGCCTTGATACCGGTCACTGCAAAGTGTTCATGGAGACC

GAGGATGTTGGCCGGACTCTTGACCTCTCTGTTCTTAATTCTTATGAAGAACTATACGGAAGATTGGCCAACATGTTTGG

CATAGAAAGATCCGAGATATTAACCCACGTGCTCTACCGGGATGCAACTGGTGCTCTTAAACAAACCGGAGATGAACCAT

TCAGTGATTTTATGAAAACAGCCAAACGACTGACTATTAAAATGGGTTCGGGCAGTGAAACTATGGGGCATGGATCACAG

GGATTCGGAATGCTGAAAGTGCACTAG

> evm.TU.supercontig_96.40 (CpARF11)

ATGGCTCATGTTGACGGCAATCTCAGAGGCTCTGCATCTTCTCGTGGGGTATCAGGTCGGGAAAGTGATGGTGATCTGTA

TAGGGAGCTATGGAAGGCATGTGCAGGGCCGCTGGTGGAGGTTCCTCGTTCCGGAGAGAGAGTTTTCTACTTCCCTCAGG

GCCATATGGAGCAATTAGAAGCTTCGACTAATCAAGAACTCAATCAGCAAATCCCTCGGTTTAATCTTCCTTCGAAGATC

CTTTGTACAGTTGTGCACATTCAATTACTGGCAGAACAGGAGACGGATGAGGTTTATGCTCAGATCACCTTGCAGCCAGA

AGCAGATCCAAGTGAGCCTCCAGGTCCAGATCCCTGTTTGCCTGAACCTCCAAAGCGAACTGTTCATTCCTTTTGTAAGA

TTCTAACAGCTTCAGACACAAGCACCCATGGTGGATTCTCTGTTCTGCGTAAGCATGCCACTGAATGCCTGCCTCCTCTG

GACATGACCCAAGCAACCCCAACCCAAGAACTTGCTGCTAAAGATCTTCATGGTTATGAATGGCGGTTTAAACATATATT

TAGAGGTCAACCTCGGAGACATTTGCTTACAACAGGTTGGAGCACATTTGTTACATCCAAGAGACTGGTTGCAGGAGATG

CCTTTGTGTTTCTGAGAGGGGATAACGGAGAGTTGCGGGTTGGGGTACGGCGACTTGCAAGACAGCAAAGTCCTATGCCA

TCATCTGTTATATCCAGCCAAAGCATGCATTTGGGTGTCCTTGCTACTGCTTCTCATGCTGTTACAACCAACACGCTCTT

TATTGTGTACTACAAGCCAAGGACCAGCCAGTTCATTATTGGCATCAACAAGTATTTGGAGTCTGTAAAGCATGGATTTT

CTGTAGGCATGCGCTTCAAGATGAGATTTGAGGGAGAAGATTCTCCTGAGAGAAGGTTCACAGGCACTATAGTTGGGGTT

GAAGATATCTCTTCCCAGTGGTCAGGTTCCAAATGGCGGTCATTGAAGATTCAATGGGATGAACCGGCAACAATTCAACG

GCCAGAGAGGGTTTCTCCATGGGAGATTGAGCCTTTCTTGGCGCCGTCTTCTGTGAATCTTGCTCAACCAGCAATTAAGT

GCAAAAGGCCCAGGCCAGTTGATGTATCAGTTTCTGAGATTACCACCAATTCAGCTGCCTCATCTCTCTGGTATAATGGA

CCATCCCAGTCCCATGAGTTAAATCAGTTAAGTAGTCCTGCCGATGTTCAGAGTTGCGACAGCCAAGTTGTCTGGTCAAC

AAGGCAGAAAGAAATTGATGGCAGCCTTGTCAATAGCAGTGGTGGATGTAACTCCAGAGTCAGGCAAGAAGGAATCTGGT

CTTCTACTCCTGTAAATGTCTCCTTGAGTCTCTTTGCGGATTCAGCTGAGGATAACAAAACTGTTCTCTCATCAAGGCCC

AAGAGTGGTGTTATGCATGACCAGGTGGAGAAAGGGAAAAAATCTGAAATTTCTGCAGGTTGTCGCTTGTTTGGAATTAA

TCTAACAGAAAAGTCTAGTGCTTCTGCTACCCATCCAGCAGTTGATTCTAGCACTGCACAAGGGCCTGTCCCGGTCACAG

ATGTTGCTGACAAGGATAGGGATCTGGAAGCTTCAAGTTCTGCCAAGGAACAGAAACAAGATACGTTAGAGGTGTCAGTG

AAAGAATCACAGAGCAAGCAGGGATCGAGTACCATGAGAAGTCGTACTAAGGTGCAAATGCAAGGGATAGCTGTTGGACG

TGCTGTAGATCTAACAGCCTTGAAAGGGTATGATGAACTTATAAATGAGCTGGAGAGGATGTTTGAGATTAAAGGAGAGA

TTGGCCCTGGTAACAAATGGGCAGTTGTTTTTACAGACGACGAGGGTGATATGATGCTTGTAGGGGATGATCCGTGGTTA

GAGTTCTGTAAGATGGTGAAGAAGATGGTCATATATTCAAGTGAGGAAGTGAAGAAGATACGCAGAAAATTCAAGTTTCC

TATGTCTTCGGTGGAGAGTGAAGGGACTGTGGGAAGCTTGGATTCAGAGCATAAGTCTGAAACATGA

> evm.model.supercontig_53.88 (CpARF16)

ATGGATTCCATTAAAGACCCAATGAGGAAAAACTCAGAGAACTTGTTAGATCCTCAACTATGGCATGCCTGTGCTGGTAG

CATGGTCCAAATTCCACCATTGAACTCCAAGGTATTTTATTTTCCTCAGGGTCACTCTGAAAACGCCCATGGGATTGTGG

ATTTGAGGCATGTCCGAATTCCACCCTTAATCCTATGCCGTGTATCAGCCATTAAGTACATGGCTGATACTGAGACTGAT

GAGGTTTATGCGAAAATCAGATTGGTGCCTCTCAGAGAAACTGGTTTTGGCTATATTGGTGATGATCACGATGTGTTTGA

TGGAAGTACTGGGGTTGATAGTTCTGATAAACCTGTCTCATTTGCCAAGACTTTAACTCAATCTGATGCTAACAATGGTG

GGGGATTCTCTGTCCCGCGCTACTGTGCGGAGACTATATTCCCTAGGTTGGATTACAACGCTGAACCTCCTGTCCAGACC

ATTCTCGCCAAGGATGTCCATGGCGAGGTTTGGAAATTTAGGCATATTTATAGAGGGACTCCTCGTCGTCACCTGTTGAC

GACAGGATGGAGTAATTTTGTGAACCAGAAGAAGCTTGTGGCTGGGGATTCGATAGTGTTTTTCAGGGCAGAGAATGGGG

ATCTTCGTGTGGGAATTCGAAGGGCTAAGAGAGGGACTGGTAACGGACTTGAATACTCATCTGGGTGGAATCCGGTTGGT

GGAAATTGTAGCTCTTCATCCGGGGGATATTCTGGTTTTTCTAGAGAAGATGAGAGTAAGTTGATGAGGAGAAATTGTTC

TGATGGAGATTTGAGGGGAAGGGTAAGGGCAGAATCCGTTGTTGAGGCTGCAACTCTTGCTGCCAAGGGACAGCCTTTTG

AGGTTGTTTACTATCCGAGAGCAAGCACTCCGGAGTTCTGTGTGAAGGCCTCAGCTGTGAGGGCTGCTATGCAGATCCAA

TGGTGTCCTGGAATGAAGTTCAAAATGGCATTTGAGACTGAGGACTCTTCGCGGATAAGTTGGTTCATGGGGGCTGTGGC

TTCTGTTCAGGTTGTTGACCCTATTAACTGGCCTAATTCTCCATGGCGGCTTCTTGAGGTGACATGGGACGAGCCGGACT

TACTCCAGAATGTTAAGCGTGTTAGTCCGTGGTTGGTTGAATTGGTGACCAACATACCAACACTCCATCTGTCCCCCTTC

TCGCCACCAAGGAAAAAGATGCGGGTTCCACAACACCCGGATTTCTCTCTACTGACCCAAGTCCCAGTGCCATCATTTTC

GAGCAACACCCTCAGGTCCAGCAGCCCCTTGAGTTGTATTTCAGACATGATACCTGCAGGCATACAGGGAGCCAGGCATG

CTCAATATGGATCATCTTTTTCAGATCTCTACTTCAACAATCTGCAGCCGGGGTTGTTTCCAGTTGATTTCCAGCAGCAG

CAGCAGCAGCAGCAGCAGCAGCAGCTTGATCATGCTGCCCCACCTTCCAGAAGCATCAATGGCAATTCTGTAAGAACCAG

CAAAATCAATGAGAGTTTATCTTGCTCGCTGACAATAGGAAATCCCACCAGTAGTTTGAAGGAGAACAGTGAAGTAAAGA

CACCGCACTTCTTTTTGTTTGGTAAACTTATTCTAACTCAACCACAAAATTGTGAAAGCTCTTCTGGGGATACAATGGGA

AATGGTTCGTTGGATGGGAATCTAGAGAAAACTACAATTTCTTCTGATGGCTCTGGTTCTGCAACAAATCAGATAGGTAC

ACAGGAAAATTCTTCGGATGAAGGATCTCCTTGGTGCAAAGATGATCAAAAAACTGATGTTGGATTCGAGGCAGATCACT

GCAAAGTATTCATGAAATCTGATGATGTTGGTCGAAATCTTGACTTGTCAGTCCTTAGTTCATACCAAGAGCTGTATGGA

AAGCTTGCAAACATGTATGGCATGGAAAATTCGGAGATGGTGGGCGCAGTGCTGTACCGAGATGCGGCAGGTTGTCTTAG

ACAAACTGGCAATGAACCCTTTAGCGTGTTCCTGAAGACAGCGAGAAAGCTAATGATTGTTCCGGATTCAAGCAGTGACA

ATGTAGGAAGGTAG

> evm.model.supercontig_49.122 (CpARF17)

ATGCCACCATCTCTACCTCCCGGAGATTTGCGCCTCGTCGATCCCCGGATCTGGCGGGCCTGCGCCGGCGCATCCGTTCA

AATCCCCCTTCTCCGCTCTAGGGTTTACTACTTCCCCCAAGGTCACCTCGAGCAGTCCTGCTCATCCCCTGCTTCTATCC

TCTCCCCTCTTGCTCTCTCCAAACCTCTCATCCCTTGCATCATAGCTGCTGTCCATTTCTTCGCGGATCCTGTCACTGAC

GAGGTCTTCACCAGACTCCTGCTTCACCCCGTCTCGCCGTCACAGTTATCTGCTCATGATTCTTCTGCAAATCAACAGCA

GCAACTAGGAGGGCGATATGATGACGAAGATGATAAGATCGTCGCCTTCGCAAAGATTCTTACGCCGTCTGATGCCAATA

ACGGCGGCGGATTTTCCGTCCCTCGTTTCTGCGCCGATTCTATCTTCCCGGTGCTCAACTACCAAGCCGAGCCGCCTGTG

CAGACACTCTCTGTTACTGACGTCCATGGCGCTGTCTGGGAGTTTCGCCACATTTACCGAGGCACCCCCCGGCGCCACTT

GTTGACCACCGGGTGGAGCAAGTTCGTGAACCACAAAAAGCTAATCGCCGGTGATTCCGTCGTTTTCATGAAGAGCTCGA

CGGGGAAGATGTTCATCGGAGTGAGGAGAGCCGTGAGATTCGGCAATGGCAGCTGCGACTATGCCAGGTGGCGGAACCAG

ATTGGTGGCGGAGTGGACAGAGTAACGGGGGAGGAAACAGAAGGGGAAGGGATAGGTAGGGAGGGTTTTGCCAGGAGCGG

TAGAGGGCGGTTGACTCCGGAGGCTGTGGCAGAGGCGGCCGAGAGAGCGGCTCTGGGACGGCCTTTCGAGGTGGTGTATT

ATCCGAGGGCTGGTTGGTCGGACTTCATAGTGAAGGCGGAGGTGGTGGAAGCTGCGCTTAGCGTTTTCTGGGTGCCCGGG

ATGAGGGTTAAAATGGCTATGGAAACGGAGGATTCATCACGGCTGACATGGTTTCAGGGGACAGTGTCCTCTTCTCAGGT

CCGGGAGAGAGGGCCATGGTCTGGCTCTCCCTGGCGAATGCTTCAGATTACTTGGGATGAACCTGAAATTCTGCAAAGTG

CAAAGAGAGTGAGCCCTTGGCAAGTTGAATTTCTGGCACCCACACCACCGCTTCAGACAACCTTCCCCCCAGCTAAGAAG

TTAAAGTTTTCTCAGAATTCAGGTTTTGTTAGTGAAGGAGATAGTGAAACCTTCTTTCCGACTGCTGGATTAAATAATTC

GACAATGGGGCAACTGAAGCCATCATTATTGAGTTATAAAACTTTTCCTGCTGGCATGCAGGGAGCCAGGCAAAATAATT

TTGGATTCTTTAGTTTAAACAACTTCATGAATGAGAATTCCTCTCAGACGTGCACTGATAATTTCTTTAGCAACAACATG

GTGGCGAAACTGAAAAGAGTATCCACTGAATTGAACATTGGTAGTCCACAGTCTGACAACTTATCACCTGACAGTCAGAG

TAGCTTTCATTCCTTTGTCATAAACTCTGTTGGAAACAGGGGATGTAACTCAACAAAAGTTGGCATTAGTTCATTTCAGT

TGTTTGGAAAGGTTATTCATATGGAAGAGCCTATTGAAAGTGGTTTTGATGGTGTTGGTTGCATCGAAGATGATAACACC

AGAGGATGCAGTGAAACTGGGGGAGTGAGCAACCTGGTAGATCTTTCCTTGACCTACCCTTATATGGAACTGCTTGACAG

GCTAGATGCCCAATGCCAAAGAGCTTCAGCTGTTGAAGCATGTTCCTTGTGA
