# Supplementary figures and images for: Genome-wide identification and characterization of auxin response factor (ARF) family genes related to flower and fruit development in papaya (Carica papaya L.)
Source: BMC Genomics. 2015 Nov 5;16:901. doi: 10.1186/s12864-015-2182-0 (PMC4635992; doi:10.1186/s12864-015-2182-0)

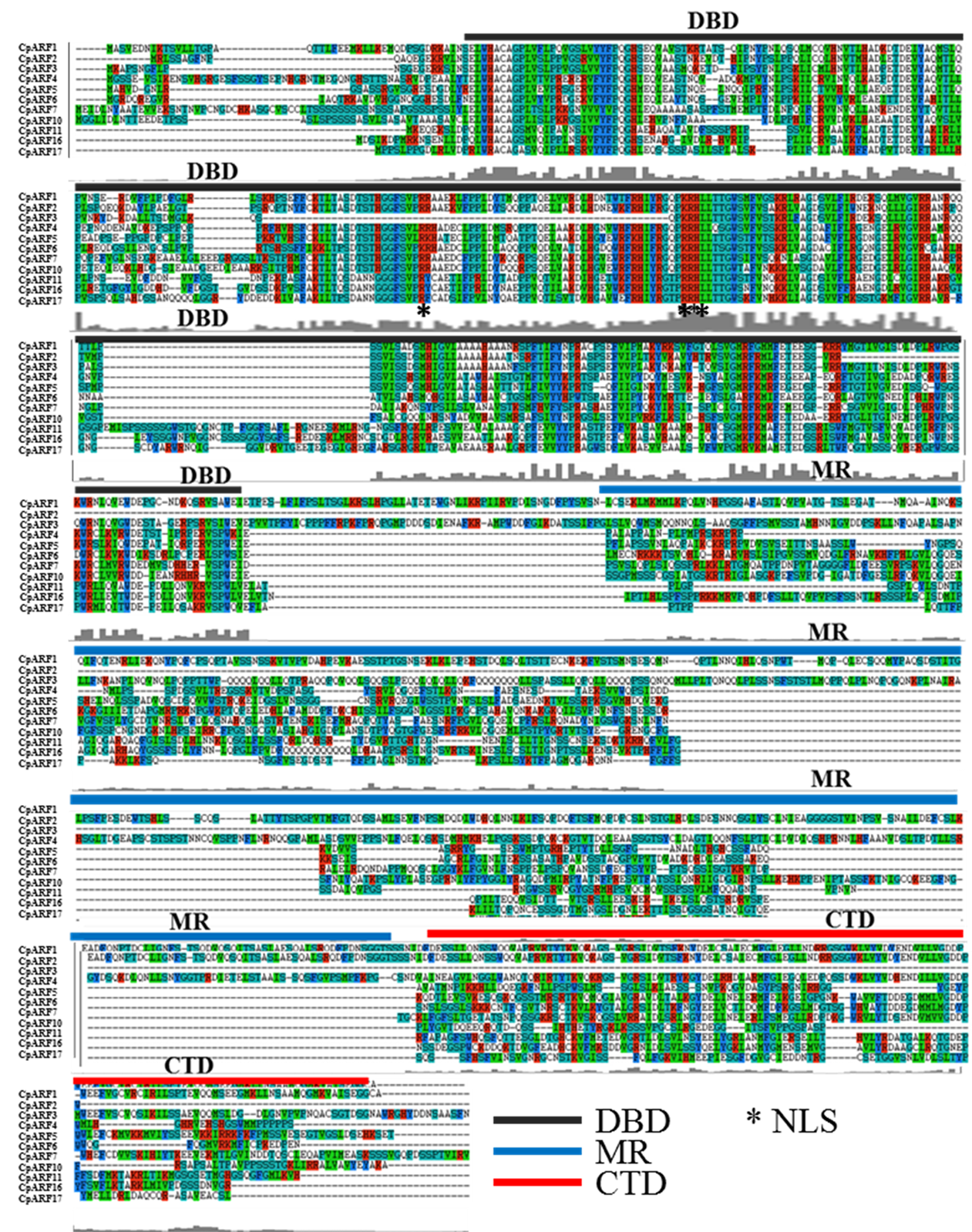

Supplement: Additional file 2: Figure S1. — Multiple alignment profile of CpARF proteins obtained with ClustalW program. Multiple alignments of the DBD, MR and CTD domains of the CpARF proteins also were showed by different color lines. Colorized shading indicates identical and conversed amino acid residues, respectively. Two NLSs were marked by black asterisks. (TIF 3259 kb) [file 12864_2015_2182_MOESM2_ESM.tif]

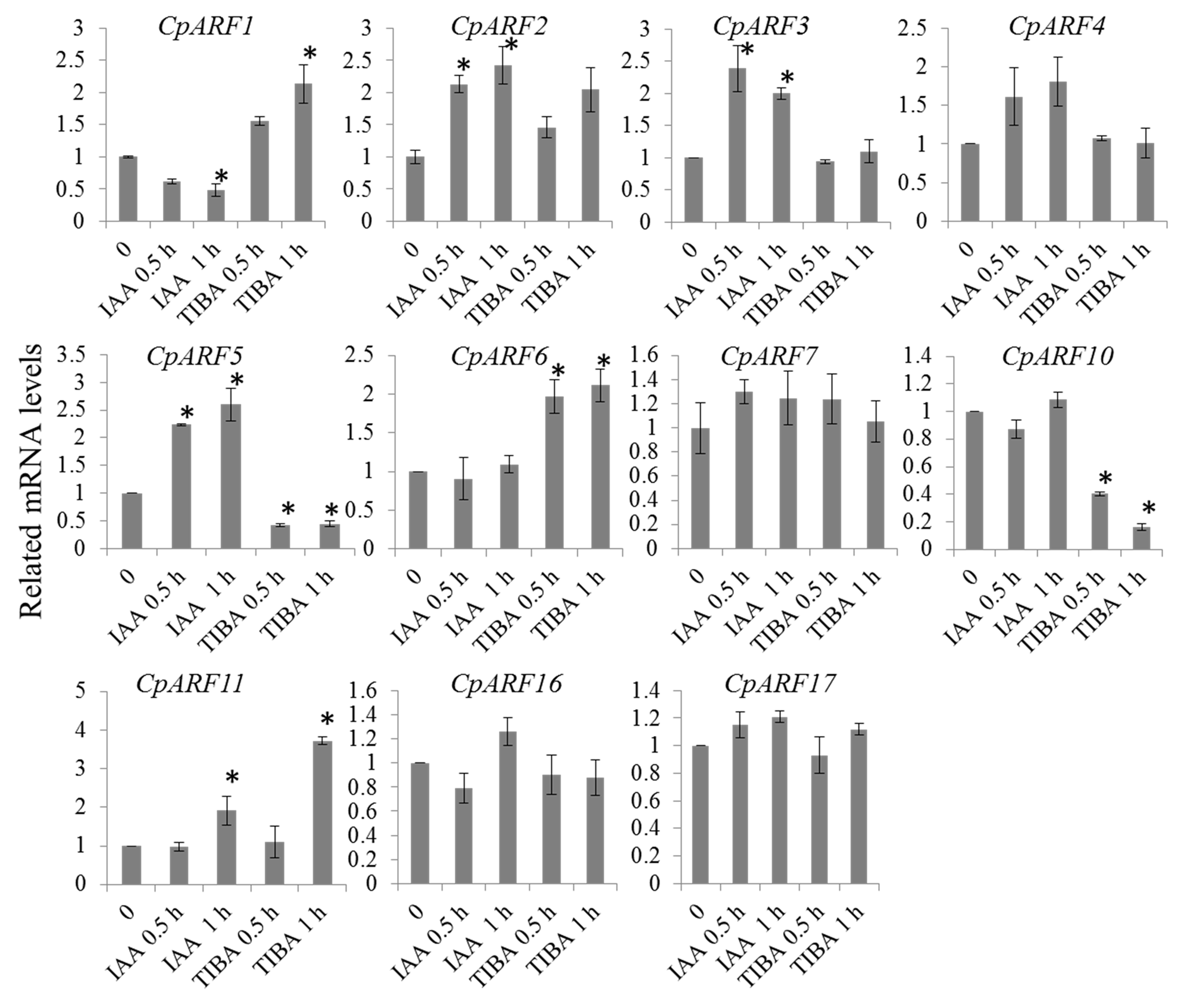

Supplement: Additional file 7: Figure S2. — The expression level of CpARF genes under IAA and TIBA treatments in flowers. The histogram shows the relative expression level of CpARF genes under IAA and TIBA treatments during different time points compared to the mock expression level. Significant (P < 0.05) differences in control and treatments are indicated by an asterisk. (TIF 424 kb) [file 12864_2015_2182_MOESM7_ESM.tif]

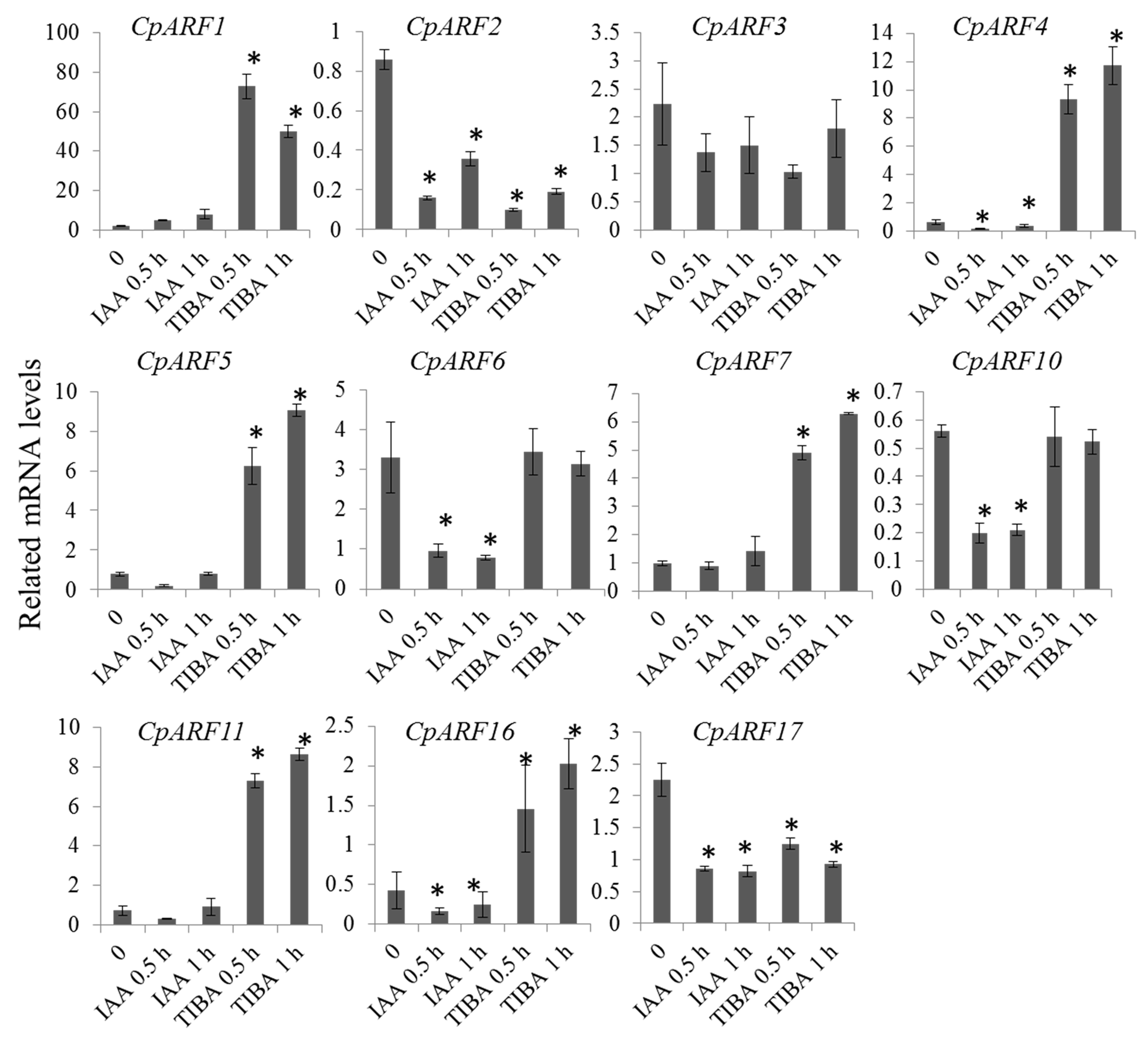

Supplement: Additional file 8: Figure S3. — The expression level of CpARF genes under IAA and TIBA treatments fruits. The histogram shows the relative expression level of CpARF genes under IAA and TIBA treatments during different time points compared to the mock expression level. Significant (P < 0.05) differences in control and treatments are indicated by an asterisk. (TIF 407 kb) [file 12864_2015_2182_MOESM8_ESM.tif]

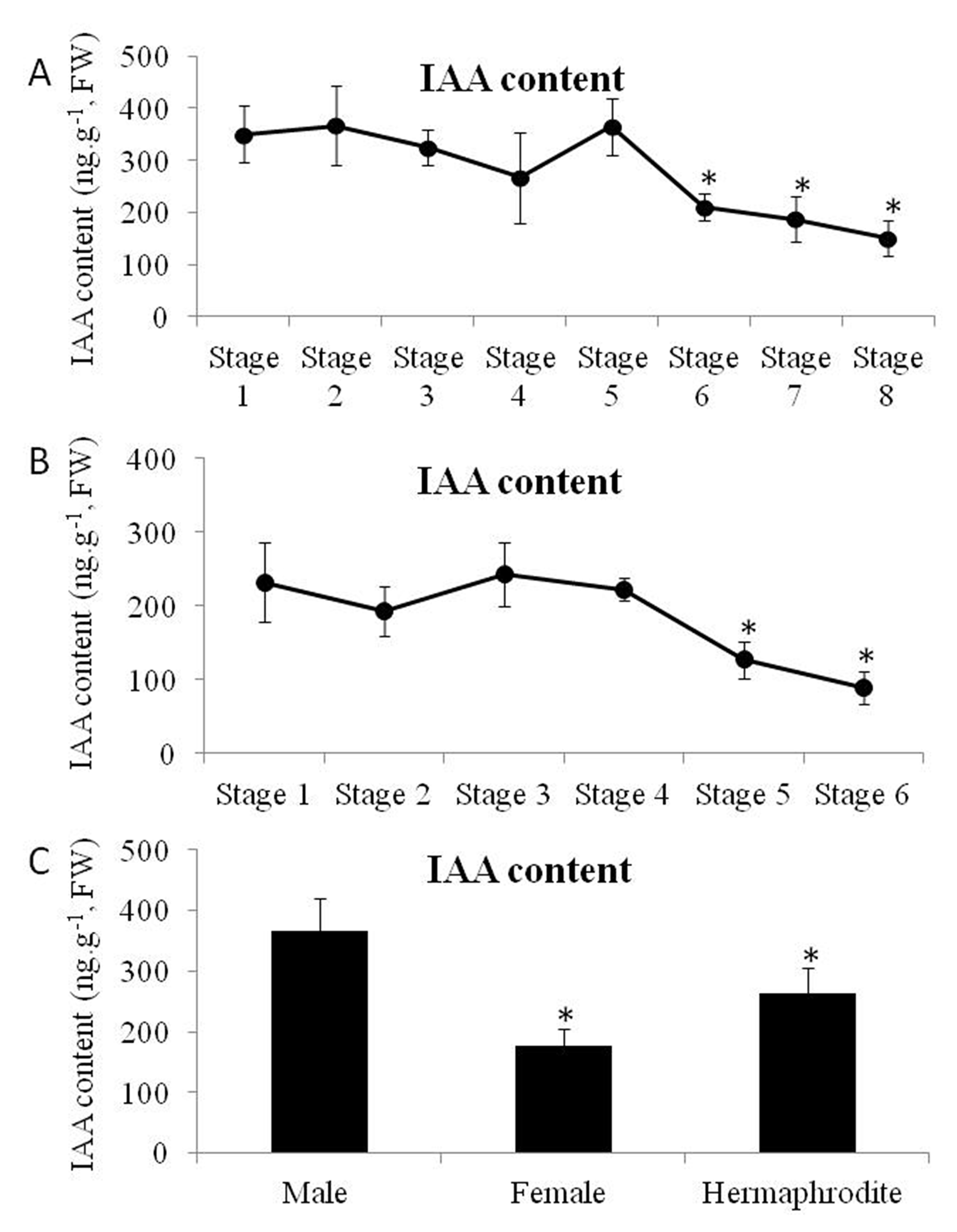

Supplement: Additional file 12: Figure S4. — The endogenous IAA contents measurment. (a) The endogenous IAA contents in the flowers under different developmental stages. (b) The endogenous IAA contents in the fruits under different developmental stages. (c) The endogenous IAA contents in the flowers of different sex types. Significant (P < 0.05) differences in IAA contents are indicated by an asterisk. (TIF 516 kb) [file 12864_2015_2182_MOESM12_ESM.tif]
