# Supplementary material for: Genome-wide identification and characterization of auxin response factor (ARF) family genes related to flower and fruit development in papaya (Carica papaya L.)
Source: BMC Genomics. 2015 Nov 5;16:901. doi: 10.1186/s12864-015-2182-0 (PMC4635992; doi:10.1186/s12864-015-2182-0)
Supplement: Additional file 3: Table S2. — Domain positions in 11 CpARF proteins. (DOCX 16 kb) [file 12864_2015_2182_MOESM3_ESM.docx]

| **Table S2 Domain positions in 11 CpARF proteins.** | | | | | |
| --- | --- | --- | --- | --- | --- |
| Gene ID | Gene name | protein (aa) | DBD (aa) | MR (aa) | CTD (aa) |
| **evm.TU.supercontig_9.161** | CpARF1 | 698 | 0-368 | 369-589 | 590-698 |
| **evm.TU.contig_31756.1** | CpARF2 | 619 | 0-390 | 390-619 | none |
| **evm.TU.supercontig_7.3** | CpARF3 | 674 | 0-379 | 378-674 | none |
| **evm.TU.supercontig_139.80** | CpARF4 | 813 | 0-406 | 407-665 | 666-813 |
| **evm.TU.supercontig_26.24** | CpARF5 | 938 | 0-317 | 318-816 | 817-938 |
| **evm.TU.supercontig_17.53** | CpARF6 | 311 | 0-311 | none | none |
| **evm.TU.supercontig_261.2** | CpARF7 | 883 | 0-228 | 229-752 | 753-883 |
| **evm.TU.supercontig_65.4** | CpARF10 | 648 | 0-379 | 380-546 | 547-648 |
| **evm.TU.supercontig_96.40** | CpARF11 | 688 | 0-357 | 358-568 | 569-688 |
| **evm.TU.supercontig_53.88** | CpARF16 | 697 | 0-387 | 388-606 | 607-697 |
| **evm.TU.supercontig_49.122** | CpARF17 | 603 | 0-381 | 382-603 | none |
