# Supplementary material for: Genome-wide identification and characterization of auxin response factor (ARF) family genes related to flower and fruit development in papaya (Carica papaya L.)
Source: BMC Genomics. 2015 Nov 5;16:901. doi: 10.1186/s12864-015-2182-0 (PMC4635992; doi:10.1186/s12864-015-2182-0)
Supplement: Additional file 4: Table S3. — Data of amino acid content in MR domain of CpARFs. (DOCX 19 kb) [file 12864_2015_2182_MOESM4_ESM.docx]

**Table S3 Data of amino acid content in MR domain of CpARFs**

|  | CpARF5 | CpARF7 | CpARF10 | CpARF16 | CpARF1 | CpARF4 | CpARF11 | CpARF2 | CpARF3 | CpARF17 | CpARF6 |
| --- | --- | --- | --- | --- | --- | --- | --- | --- | --- | --- | --- |
| Ala | 4.008 | 5.3435 | 3.5928 | 2.7397 | 5.2133 | 5.4054 | 7.5829 | 6.1135 | 5.4237 | 4.5045 | 0 |
| Cys | 2.004 | 1.3359 | 1.7964 | 1.8265 | 2.3697 | 1.5444 | 1.8957 | 0.4367 | 2.3729 | 2.7027 | 0 |
| Asp | 5.2104 | 4.9618 | 4.1916 | 5.4795 | 4.7393 | 3.4749 | 6.1611 | 5.2402 | 3.0508 | 4.5045 | 0 |
| Glu | 6.4128 | 3.2443 | 5.988 | 3.1963 | 4.2654 | 5.0193 | 5.6872 | 4.8035 | 4.7458 | 5.4054 | 0 |
| Phe | 4.008 | 4.0076 | 4.7904 | 4.5662 | 4.7393 | 5.7915 | 1.4218 | 2.1834 | 4.0678 | 8.1081 | 0 |
| Gly | 5.4108 | 4.771 | 6.5868 | 6.8493 | 7.5829 | 8.4942 | 5.2133 | 7.8603 | 12.881 | 8.1081 | 0 |
| His | 1.4028 | 1.145 | 2.994 | 2.2831 | 3.3175 | 0.7722 | 1.4218 | 3.9301 | 1.6949 | 0.9009 | 0 |
| Ile | 4.4088 | 2.8626 | 3.5928 | 4.5662 | 4.2654 | 5.7915 | 2.8436 | 1.31 | 3.7288 | 2.7027 | 0 |
| Lys | 3.6072 | 2.8626 | 4.1916 | 4.1096 | 7.109 | 4.2471 | 6.6351 | 4.3668 | 3.0508 | 4.0541 | 0 |
| Leu | 8.2164 | 10.878 | 11.976 | 8.6758 | 7.5829 | 6.9498 | 6.1611 | 8.2969 | 4.7458 | 8.5586 | 0 |
| Met | 3.006 | 3.0534 | 0 | 1.3699 | 2.3697 | 1.1583 | 0.4739 | 3.4934 | 1.6949 | 2.7027 | 0 |
| Asn | 6.4128 | 6.8702 | 4.7904 | 6.3927 | 5.6872 | 7.3359 | 4.2654 | 3.9301 | 3.3898 | 8.5586 | 0 |
| Pro | 6.8136 | 9.7328 | 4.1916 | 8.2192 | 9.0047 | 8.4942 | 5.6872 | 11.354 | 8.4746 | 5.4054 | 0 |
| Gln | 7.4148 | 13.168 | 7.7844 | 9.589 | 6.1611 | 6.1776 | 6.1611 | 2.6201 | 5.4237 | 4.955 | 0 |
| Arg | 2.4048 | 3.2443 | 5.3892 | 2.7397 | 4.2654 | 5.7915 | 3.7915 | 4.8035 | 6.1017 | 2.7027 | 0 |
| Ser | 15.03 | 11.26 | 14.371 | 15.982 | 12.322 | 10.811 | 18.483 | 15.284 | 14.915 | 13.063 | 0 |
| Thr | 7.4148 | 4.9618 | 8.982 | 6.8493 | 1.8957 | 5.4054 | 4.7393 | 3.9301 | 5.0847 | 6.3063 | 0 |
| Val | 3.8076 | 3.8168 | 2.994 | 3.1963 | 5.6872 | 3.4749 | 9.4787 | 5.6769 | 6.1017 | 5.4054 | 0 |
| Trp | 1.8036 | 1.5267 | 0.5988 | 0.4566 | 0.4739 | 0.3861 | 1.4218 | 1.7467 | 0.339 | 0 | 0 |
| Tyr | 1.2024 | 0.9542 | 1.1976 | 0.9132 | 0.9479 | 3.4749 | 0.4739 | 2.6201 | 2.7119 | 1.3514 | 0 |
