# Supplementary material for: Genome-wide identification and characterization of auxin response factor (ARF) family genes related to flower and fruit development in papaya (Carica papaya L.)
Source: BMC Genomics. 2015 Nov 5;16:901. doi: 10.1186/s12864-015-2182-0 (PMC4635992; doi:10.1186/s12864-015-2182-0)
Supplement: Additional file 10: Table S7. — Promoter analysis (locations from ATG) of the genes involved in flower development and fruit ripening. (DOCX 16 kb) [file 12864_2015_2182_MOESM10_ESM.docx]

|  | | | |
| --- | --- | --- | --- |
| Gene | AUX1(TGTCTC) | AUX2(TGTCYS) | Function |
| evm.TU.contig_32595(CpFT1) | -1953 bp | -1442 bp | flower development |
| evm.TU.supercontig_32.19(CpFT2) | none | -1125 bp | flower development |
| evm.TU.supercontig_107.28(CpFT3) | none | -808 bp | flower development |
| evm.TU.supercontig_13.181(CpLFY1) | none | -1459 bp, -1312 bp, -1029 bp | floral meristem development |
| evm.TU.supercontig_1.162 (CpAP1) | none | none | floral meristem determinacy A class |
| evm.TU.supercontig_55.116(CpAP2) | none | none | floral meristem determinacy A class |
| evm.TU.supercontig_184.7(CpAP3) | none | none | floral meristem determinacy B class |
| evm.TU.supercontig_26.316(CpPI1) | none | none | floral meristem determinacy B class |
| evm.TU.supercontig_50.73(CpAG1) | none | -1058 bp | floral meristem determinacy C class |
| evm.TU.supercontig_414.3(CpSHP1) | none | none | floral meristem determinacy D class |
| evm.TU.supercontig_14.222(CpSEP1) | none | none | floral meristem determinacy E class |
| evm.TU.supercontig_43.78(CpSEP2) | none | none | floral meristem determinacy E class |
| evm.TU.supercontig_660.1(CpSEP3) | none | none | floral meristem determinacy E class |
| evm.TU.supercontig_3.196(CpSEP4) | none | -1445 bp, -435 bp | floral meristem determinacy E class |
| evm.TU.supercontig_84.52(CpETR1) | none | -440 bp | ethylene signaling |
| evm.TU.supercontig_151.32(CpETR2) | none | -1133 bp, -1084 bp, -231 bp | ethylene signaling |
| evm.TU.supercontig_5.78(CpCTR1) | none | none | ethylene signaling |
| evm.TU.supercontig_128.50(CpCTR2) | none | none | ethylene signaling |
| evm.TU.supercontig_9.380(CpACS1) | -1257 bp | none | ethylene synthesis |
| evm.TU.supercontig_117.57(CpACS2) | none | -441 bp , -244 bp | ethylene synthesis |
| evm.TU.supercontig_132.27(CpACO1) | -1295 bp | -522 bp | ethylene synthesis |
| evm.TU.supercontig_64.148(CpACO2) | none | none | ethylene synthesis |

**Table S7 Promoter analysis (locations from ATG) of the genes involved in flower development and fruit ripening.**
