# Supplementary material for: Genome-wide identification and characterization of auxin response factor (ARF) family genes related to flower and fruit development in papaya (Carica papaya L.)
Source: BMC Genomics. 2015 Nov 5;16:901. doi: 10.1186/s12864-015-2182-0 (PMC4635992; doi:10.1186/s12864-015-2182-0)
Supplement: Additional file 11: Table S8. — The promoter sequences of reproduction-related genes. (DOCX 28 kb) [file 12864_2015_2182_MOESM11_ESM.docx]

**Table S8 the promoter sequences of reproduction-related genes.**

> evm.TU.contig_32595(CpFT1)

ATGAGCCAAATTCAAATAATTGTGGATCTATTTGTGAAATTTGTCTCCAATATGTCCCCCAAATGACCATATCATTAACCTCTTTACGTCTACCGTATCA
TAAATCATGCATGCAAAAATGTTTTTTCATGCCCCTATTTTATTTGGAAACGTTGCTATTTAATGCTGCTATTTAATTTGGAAAGATGATCTCTGGATTA
TGTGTCAAGGGTTACACATTTTTAAGCTTTGTAATTGGTAAAAATGCTTTGCAAATAATTTTTAGGTAAAAATAAATTTAAACGGAAAAGCAGTCAACTT
TATATTTTAAATACTAAATTTTTTCTTATTTATGATTAATGTAAAAATTATAATTTATTTTATTTGTTAGTTTAAATTTAAATAAAATTTATTTTTTAAT
TAAATTTATTTTATTTTTATCTTTTAAATATTATGTAGTTAATGAGAGTTTATCATTTAAGATATTAATTCATATTTATCTTTTAGATAATGATAATTTA
TTTTTAAGAGATTAATTTATATGTAAATATATTTAAAAATAAATTAAGTTATTAATTTTATAGAGTTAATGAAAATTTAAATTTTCTTTATATAGAAAAA
CTCTAACATATGCGAGAAAAAATTAATTGAAAAGGTAAAAAAAAAAAAATTAACACTAAGGATGTGGAAAAGTAATTATCAATAGATTTTGCACAGTTTG
ACTGGAATGAACTACTTCATAGGAGGGACAAATTAAAATTTTATATCATACAAACTTCATTATGTAATTAAGTATTAATGTAGAAAAATAATTAACACAA
AATTTATGTGAGTTAATTAGGATGAACTGTTTCCCAGGAAGGATAAATTCAGATTCATTATAAAAGAATTAACAAGTAACATAGAAAACTAATTAACACT
AAGGATGTCAAAAAGTAATTATTAAGAGATTTGCATGTTTTGATCGGAATAATTCATAAGTATTTCAGAGGAGGAATAAATTCGCATTTATTAGCAAAAA
CAGATTTATGTGTGTGATTTAATTAGAACCAACTATTTCTCAGAAGGGATGGATTAGAGGTTTTTACTAAAAAAATTTAGAGGTAATGTAGAGAAATATT
TAACACCAAATCATTTATATGGTTTAATTAAAATGAATTATTTGAGAAGAGTAATAGACTCAGAATCCTTATAGAAAGATCATATGGTACATGAAGTAAA
GACAGAGAGTTAATTGTGAAATTTTGTATTAGAGCAGTAAGGAAAAGATTCTCCAACGTAGTGTGCTCTCCACAAAATTTTTGGAAGAAGAATAAAATAA
AAAACAATTGGTCCAGTTGGGTATCAGCAATGAAACTGTAGGACATTTTGGAAGCGAGGAATAAGAATTGTTTTGGAAGAGAAAGTGATAGTAGGCAGAA
TCTGTTGATGTTACAGAAAGTTAAAAAGTAAATATATACATACATATATAAATACGTGCATAAATAGAGGTGTACTCAGCTGTGGTTCATTTGAAAAACG

> evm.TU.supercontig_32.19(CpFT2)

TAGTTTAATTAAAATAATAGACGATTTATATAGCAAAAGAACAATTTGTTTAAAAAACAAAGTTGGAGTATGTAGTTTATATTATCAAATGGGATCAACA
GAAGAATATGAAGAAAAGAAGAAAAAGATTTTCCAAATATAGAAACCTCCATTCTCTAACATTACTACATATATATATCTGTACCATACATAGTTGAAGA
GAAGTGAGCCAACAATATAAGACCTCCCTCTATAAATCTTGAAATGACCCATCTCCACAACTGCATATAATATATGTCTGCATGCATGGCACGACTGTAA
TGGAGTTCCTTTATGCCCTGCAGAACAATTCCTGTTGCATGGAATATCTCTAAAACAGGAAAGTAGCTGGAATGAAGGTTAAATTAGGAAAGGTTCCCAC
CAGCACCCATCTTCAGGAATTTTCTCTTTCTTATATCTTTAACCCAAAGGTCCTTTGTGCTCTCGCAGGATCTGCTTCAATTCTCTTTAAGCTAACACAT
ACTAATTAAATAATCACATTATATACATGTGTGTGTGTGTGTGTGTGTGTGTTTGTGTGAAAGAGGTAGCTAGAGAGAACTGGTTTATATATGTATATAT
ACACTATTATATATATATATATTTATATATGGGTTTAATTTATACATGAAGTTTTCCAGACCTTATCTAGGGTTATATCTATTTCTATGTTATTTATGCT
TTAGTTTGATTTATGACTAATATACATGACTTTCTTTCTTACCTGTACAATATGTACTCTGAGTAATCATAAACAATCATTATCTCTAGAGTAGTAGAGC
CAGATCCATTAATATTCTTGAAGTATAATTTTTTTTCCTTGAAAAAAACATTCAAAATTAATTTCTTTTATCTATCAAATTATTTTCATATATCATAGAG
TAAATATGAATATTACAAATTTTTTCCCCTCAAATCCTAACCCCACTACACATTAGAACATAATATTTATGTCAGATATGCTATTAATCTTACCTCTATA
AACACATGATTAACGATATATACATATATATAATTTTCTTTAAGTTTTTACAGTGGTGAATATCTTAATATCAAAATTGAATGATGAAGGAGGGTTGGAA
ACATGCCACATAATGTAGTAGGGGCCGTTCTCAGTCAAAATTTGAGGCTGTTAAAGCGGCTATCAAATGTTTAGTCTAATTTAGCGTGCGCGAGTATTAC
TATTTAGAGGACGAAGAAATTAAAAAGAAAAGAAAAGAACCCTATGCATATATATACACACACATATTATATATAAGCACTAATTAGTTAATTAATTAAT
TAACTTATGTTCTTGTCTTGATATTCCACTAGCTAATTTCTTAACACCCCACTAACCTTTGAAGTTCCAAGAATGTAAATATATGTTTATAGGGTTCATC
AACACTATAAATACCTGCAGAAAGTCCAAGAAGAAATTAAGTTTAATCGATCACAAGCGATTTTTGAGCGTTCAGAAGTGTTTTTCGATCGATGGCAAGA

> evm.TU.supercontig_107.28(CpFT3)

CGTATGTGCGTACGTGATTCTCGATTTTGCCCGCTAATATCTATGTGCATGCATGTTAATCTTGAGTTACCATTTATAGTGCATATGTTCAACAATTTCT
GAAATGTAGTAAAGATGAGAAGGAATTAAAGTAATGAAGCAAAGAAAAGAACAAATTAAAGGGTGATATGATATGTGTTTCGGCTTTTTATAAAAGACTG
TTCTAAAGCACCCAGTGAGGTCTCCTCAAACATAAATTCACATTAAAACTTTTGTTATCGTGCATCTTCAACACTTGAACTTAAAATTTTTAGCCTCTTT
CTCGAAACTTTTGTTAAAAAAAACCTGTATAATATATATATATATCATGCAAGAAAAAAAAAACAAATAAATATCAATTAATAAACAGGAGAAGAAGATC
GAAGAGGAAGGAGAACCAGCCTGCCTGTAATATAAACACACAGTATTTTAATAATGTAGAATCTAACTTACAAAGAAATATCAGGCATAGAGAAAAAGAT
TGAGGGGCAGCAGAGGAAATAAATTAGACGAGCCATGCATGCATCAAGAGAGCAGAGATGAATGGTTTATGTAAAAGCAATACGTATACGTATATGGTCC
ATGCCAAACCAAAAATAAATATTTTGTGCTCAAAGTTTTGTCTTTTTTCGTCGTCTCAGTGGACGATGAATTGGTGGAGAACAACTTTTGTTGTCCCAAA
CTTAAACTGGTTGCTATAAATAGTAAAGGGATCCTTGCGTATTATTGGAAAGTAACACAGATATATATATATATATATATATATGTGTGTGTAATTATAT
TCGTCGAAGTTTTAAAATTACATTTGAAGGCAACCCACCGATAATATAACATATATACATACAATTCCCACCTTCACTTAATCCTCTAGTGCTAAACGTA
CCAAATCACCCTTTCTTTGACAGGACATGTTTTTATTAAATCATTTAGCACATCACAATTCTAAGGTGAAGCACCTTAAAAGCCAAATTCCCATTTGCCA
AAAGATATTATATTATATTAGGAAAAAAAAAAAGTTATATTCTTCTTTCTCATCATTATCAACTTAAGACGTGTTAGATTCTATATTAGGTTAATAATTG
TATATGTATATATAAAATAAAGTCTACAAGCTTTTTGTCAGACAATGGATATCTTACCGCAAACACGTACTGACTCACTCCATAAAATTCTTAATCAAAT
TCCACCTCTTCCATTTGATACTCTCTAGTGCCTTCATTCCACATTTATGTGTATATATATATATATATATAGCACGTGTCTTTTCCCTCAATAGAATACG
ACCCTTCATCATATATATGTACATGTGCATGTTAATGAAAACATAACCCACTTCAATTTTGAGCTTCTAAATTAAGCTCTCTCTCATTAATCATTCTCTC
TGCCTATAAAAAGCTTGCTCCTCTTGCTCTCACCCAAAATACTAATAAGCAATTTACGCTCATTCTTGTGCTGTCGTTTTGGAGAACTGTGTGTGCATTA

> evm.TU.supercontig_13.181(CpLFY1)

AGCCCAACTGACTGGAACACCTGATGGCTTTTATGTACTTTGTCTGCAACTTTTTTTTTTTAAAAAAAA
ATTTATTTTTCTTGTAATATTTGTCAAGGAATAGTGATTTTATTTATTATAATTTATTTGAAATGTCATTTTCAACGCTTGTTAATGTATGAAATTACAG
AATTGCCCTTCGCGACCCTGTCTGTCTTAAATGAAGCTTTGCTGTTTACAGATTTCACCAGAGTTTCAGGCACGGAAACACCCTTTTACAGAACCTCAAT
ATATTAATATATAATATAATGAACATTTATTATTATTATTGTTTCAGAATTCATATATTTTTCTTGAAATATATATATATATATTTGGCAGGAAGATAAG
AGATGATAGATGATACAAGTAAAATTGCTAATGATTTTAGATGCATACGTATCGAAATGTGAGTGGGTAACTGGATTCACATGACCCCCCAATTCAAAGA
ATGTCCCTGCAAACAAACTATAGATTTCTGACTCCTTTGCTTCCTCCAACTGTCAAAGTCCCAACAAAGCATCATAAACCCACTACTTCCCTCTTCTCTC
CCTTCGATTTCACTCCCCTCCACATATTATATACATATATATATGTATATATACCCGACCCAGTTGATCCTGCAAGGGCAGTTTTGGAATCCCAAAAAGT
CTGTCTTTATATTAGTAGTAGTAGCTGCTAGCAAACCCCAAACAGCCAACAATTTGCCAACTCTTCCTCAAACAAGTGTCTAAACATAGAGAATTGCTTA

> evm.TU.supercontig_1.162 (CpAP1)

CATACGTAGCACCAACATTATGCACCATGGTATGAGATGGGTAAAGTTTAATATCAAATTCATGCAATAAAAGTAGAGTCAATTTGGAATTTGGGACTTG
AGATGTACAATTGATACGAGATAATATCATCTTTAGTAGTGTAGTACCAAATTCATTAATTAACCTATGTAAATTTTGATTGTTACAAACGGTAAGAAAT
TATGTTTTCTATAATAAATAGCATATAAAAGTAAATTTTGTTAAAAATTAATTACCAAAACAATATGACAGTAAAATAATTGAATCTTTTACTTGAAGAA
AGGAAGAAGTAGTGCTTGCAGTACTTTCTAATCCTTTTTAAATTACCATCATATAGAGCTTAGTAAATTATTAATTATTGTATATTAGTTACAAGTTAAT
GCTCTTAGAATTCTTTTGAATTGTGCAAAATTTTATTTAATATACAAAAAAAGTGACAAATGTAACAATATTTTATTTTTTTATTAATTAATCGAATATA
TATATATATATATATCCTTATATCCTAATAAACGATATACAATATGTATAGAATTTTCTCTAATGGGACATTTTGTTACCTAATAGTTAAAGAAAAATCT
TAAATTAGGTGTAGATAGAAAAATTTCTATATCTGTTCATATTCGTAAAAATATTTAAGCCAATAATTTGAACGAAAGAATTGGAATTAAAAAAATAATA
TCATATTATATAATTAAACATCTCTATAAATATAGAAAGACGAATTAGAAAAAGAATTAAAAAGGAATATCGAATGTTTTCTTTGAAAGCAAACAATTCT
CTCTATCAAAGGGTATTGAATCATTGATATATTACGATCTATATAATATTCTATGCTAATATTTTAATTGTACTAGCTAGAAACTAAAAGAAATACATAT
ATATATTTATATAAATGTGTAAAAATTTGAAGGCTTGATGATCTAAGATTTAATTCCTTGTAAAATCTCCGCCGTCAAGACAAAGCGAACTTGACACTTG
GGGTTCCTGACACGCTTAGCATTATGAGAACCCTGTATTTGAAAACACTTTAAAGAAAATAAAAATACAAAATTTGTGGTTCCTACACGGGCCAGTGACT
GATAGCAAAAGGACAATGAGCCAATCACAACTCGACAGCACATTCAGTGAAAACCACTTGGGTTTCTCCAAAAAACCACTAATTTTCCCCTAAAACAGCT
TTTTTCTTATCTTTGAGAATTTTTCCTATTTCTATGTGTGTATATATACACACTTACATGCTTCCTCAGACACAAGAACCTCTCATTTATTTTAGCAAGA
ACAAACACAAGAGAGGAAAACAATTCCCCATTTGGGCTTTTTAATACACTACCCAAAATATCCATTTATATATATATATAAATAGTTTTTTGAGTTGTGT
GTGTGTGTGTTATAAGTTGCAAAGCCTGGTTTTGTTTTCCTGGTTGTGTTTCTTGGGTTCTGGTTGTTGGGTGTTTTTAGAGAAATCAAGAAGAGTAGAG

> evm.TU.supercontig_55.116(CpAP2)

GTACAAAGTAAATTATTATTTAAGAGATATTATGAAATAATAGAATTAATTATAACAATTGAATGTTTTTGTAATGATAATTAATAACTTTTGAAGGTAA
AAATATTATATTATTTTTTAAATTTTGATATTAATTATATTCGTTCGTACTATTAACTACAAAGTAAAATATTATTCAAAATATATATTATAAAATAATA
AAAATGGCTTTTAATCGTAGTTTTTTACATTTTTAACAAGAAAAATGTTTTTGTAATGATAATTAATAACTTGGAAAGCGAATTTTTATAGTATTATATC
ATCGATTATATTCATTCGTACCGTTAATTATATATAAAATTTTATAAAATTTATATTCAATAAAATATCAGTAAGTTAAAAAAAAAATAAAAGTGAAAAG
TAAAATTGCGATATATTTAATAATTTTTTTTTTTTTTAAGGGTGAAGAAAATGAAAGGGGTAGGGATAATAATCATGCGACCAATAATGGTAATTGAGTA
ATAAACCATCTTCTCTAGTCTTGCCGCTGTCTATTTCCATTAGTGGGTAATGTAGAGAGAGAAAGCTAAGTTCCCAAATCCATGTAAGAAATATATATAT
ATATATATGTGTGTGTGTGTGTGTGTGTGTATATACAATGAGAGAGAGAGAGAGAGAGATTGCTTTTCGATTTGGTTAACGTGGGAAAGTAAGGTAAAGA
AAGAGATGGTTCTTTCTTTCTCTCTTTGTATTTCCTTTTTCTTTTAAATGAAAAAACTCCATTTTTAACATCTAACAAGTCATGATTTTCGTCTCAGGAA
AACCGCCAATGAGAATATAAAGAAGATAGATACAGTAAACCCTATAATAAGTATACAAGGAAAAAAAAAGATTACCCAGTAAAAAAATTTCTTAAAAGAA
AAAACAAAAAAAAAACTCATGAATATTATGACAGGAGAATAATTTATAGTTTGCAAAAATTTATAATTTATTATAATAATTAAAAGTGAGTGGTTAGAGA
GAGAAAGGGAGAAGGCATAATATGTAGAGAGAGGAAGAAGAAGTAAATAATAAGAAGGGAGTTGTGGATGCTTTCAGCTACTAGCTCAAACTGGCCTCGT
CTATGCTGTATTGGGCAGAGTCTGTAATTCGTAGAGATCTTCAAAAACATCTCCTTTTTTTGCAATCTCTTTCCTTTAGTCGTAGGTTTTTTTTTTTTTT
TTTTTTTTAATTTTATTTTCATTTTCTTTCTATTTGTATTTTCTTTTCCTTGTTTACTTTCTCTCATTTGAATACTCGACTTTGTCTTCACGAGGACTCT
CTTTTTTCTTCTTGCATTCATTGAATGCCCGTAGAGGACGAGAGATCGAAGTAATTGTTTCTCCTCTCCGTTTGTTTGTTTCCCCAGAAAAAAATATTGG
TTCTTTTGGGGAAAATTTTGTTAAAGCTATGGTTTTTAAATTGGGTATTTGAAGAGGGTATATCTATATAAATATTTTGAAGGTACAGCAAGAAAGAGAA

> evm.TU.supercontig_184.7(CpAP3)

GCTGGAAACAAGGTATGTGATTAAGGTCATGATGAGTGAGAGTATTTTCTTCCTTCTCTTTTTTCCGAGATTGTTTTTTCTCTGTTTGTTGATCTGTTTT
CTACCCGCCATCTGCGGTGTTGGTTCAGTTACTGATGCGTAATGCTTTTTAGTTATGTGGGGTTTTTGATGTTTGATTGATGGGTAAGCCCAGCCTAATA
TATATATATATATATATTTTTTTTTTACAATTTCTCGCTACCTAACATTATTATTTTCTATGAAGTCATCGAACATTTTTAGGGTTTTGATGCTGAAACC
GTGAAAAGATTGTTTCTGCGAAGAAGGACGCAATGTTGTTTTTTAGCACATTCGTCAGTGAGAAATCTGGCGAAAAATGCACCCTTCTTTTTCATTTCTT
GGTGTTATTCTCTAGATATCCTGTTTTCCTTCAGTGGTAACAGAGTAGTGATTCACATTTTCTTCTTCAGTTTGTGAGGTGATTAAGGATTTTAATCCAT
TTGTGGTATTGCTATTATGTTGTTGATCGATCAATTTTTCTACCACTAATCAATGCTTTTGCATTTAATTTCATATACTGTTTTGTTTCTTGTCATAATT
TGATTATATATATCTCTCTCGTTTGAGTACGCATCTAATCTTGTTGATTTCCTCGATTTGATGCAATCACATCTGGGTACGCAACCGATTTCTTTAGGAA
CATAAGCTCACATGCGGTTGTTTTATATGAGTCTGCCTTCTGGTTATGCATGTATTGATTGAACGAAACATGGGTTGAGTTTGTCATGGATTTTACTTTC
AGTTCTCAAGTTTGGGATTTTGATTTTCCCATCTGAAAATAGGTTTTTTTTTTATGTCATTTGATATAACACAGATTCCATTTTGTCCTCATTTTTTATC
TGTGAGAATGAATTAGGCTAGATGAAATCAGGTTTAATGTCCTTCAATGTAAAGTCTAATGATTTTTGTTGCAGGGTTATGGTTTATCGCTTGTAATGAT
TCAGGAGTCTTGCTGACTGTAAGATCTTTCTGATTCCTGTAAGCACATTGGAAAAATTTTGTGGCATCATTCTATTGTTTTTTGTTTTAATTTTTTTTTA
TTTTGTTCTTTCGACTATGGATATCCTTTTAATGTTACGGGAGAACTGGTGATTCACCACACTATTTCTGATGATGCTACCTCCAACGTTTACTTGTTTA
TCTCTTTTACGTGTTTGGCAGATTAGGTTGTAGTTTGGTCCTTGTATTTGATTGTCAACTGTTTTATGTTTCATTATCCTTCCTTTTTTGATGCTTCACC
GCTTGAGTCTATATTCGCCTAAGCCTTGGTCTATTTCTCCATGTTGGTTCTAACATCTTCATAGATGATTCTATTTTCACAGGTCTCACGTCTAGTAACA
AAGCATTATGATCTCATCTCTCTTTTATATTTAGATTATGTTTAGTTGTTTTTATTTTTCTGAGAGCAGTTTAATTTTTGTTACAGGAATTTCTTTAATT

> evm.TU.supercontig_26.316(CpPI1)

TTACTACCACAAAAAATAAAAACTTTGTATAAGTTTTCCATCTCTACATGCAAACTTCAAATTCGTCGGGGACAAAAATTTCAATGGAATTTTTATCATT
ATTTAACTCCCAATAAAAAAATATTTTCTTCTTCGTAAAATCTAAATATAAATGAATTATCTCGTTTAGATCTTAAATGATCTAATGTAAAATCACCGTT
CATAGGATACCCTTCCTTTTGAAGATCATATCTATTTTCTTATTTTGTCCTTTTGTTGTTTTCGTCATCTATTTTGTATTTAATAATAAATAAATAAATC
TGGTTATTTATTTGAGATGTAGCAAATGTAGTCAGTCAAATTAATAATATTTCTATAAAAATTTAAACGAAAAATTTAAAATTAATTAAAAATGTTTGAA
TTTTAACGTCTTATTCCTCATTGAAATTAGTTTTTCTCGAGTTATTTTTCTTTCTTACACACACGTAAACACACATTATCTTTCTCTCTCTACACGTTTT
TCTTCAAATATTTGTTACATTGACAATCATCTTGTCGGTATTAATTATGAAGTACAGGAGTTCAAGGGTTTGAAACTTGACATTTCTTTTTATTTATTTA
TTTGTGATTTTATTTTATAAATTTGATAGAAGAGTATGAATTATCTCAAATCTTATTAAAAAATAATTAATTTATCGAAAAATTTTAAATTATATATTTT
TTATATTAATTTAATATATTACAATTTTTTTTACGAGAGTGTTTCGCGTGAAATGAGAATAATATTTTTCCTCTTTTTTAATCATATGTGCAAAATAAGC
CAATGAAATTCTAAATTTAATTAGTATGTTTATAGCTTTTGACTCTTGGTTAATTTCTTAGATAAGGCAAATATAAAATTCACATATTCAAAAATTATCC
TTCTTAATTTAAAATTTTATATCGGTTGTATGTAGGATAAGTCATGTTTATAATTTAAGTAAAACACAATTGATTGTGATTTTATGGTTAGGATAAGTCA
TGTGTATTTTACCAAATTTAGTTCCATTACTGAAAACCCTAGCTAGACATGACCATCACAGACTTCAAGGCCAACTCGTGTCAATCAGATCATGAACACA
TTTCATTTTACGTATCAAAGACATCCTAGAGTTTCTTGTGTAATTCCCATTGTTTTTAAAGCCATACCGCACAAGCCACCAACATTTCTTAATCCAAACC
CTAAAAAAACCTAAACTCCCTATCAATCTAATATCAGTCACAGAAGACAGATATTGAGAGAGAGAATCATAGGGACCAATGGTTAAGAAGGAGAAATGAA
AAAAGCAGTTGGCTTAGTTGTGTCAATAAACCCATAAACCAACCCTCCCTTTCTGTGCCACTAAGAAAGTTGTTTATCTTCATACTCATAATAGAAATAC
CCTTCCTTCTCTTTCACTTCCCTTGGAGACTTTATTTGATATCTTCTCCGTACAAAACAAGAAGAGCTCAGAAGGAAAAAAACACAGTGGGTTTGGTGAA

> evm.TU.supercontig_50.73(CpAG1)

GACTCACTCTTCACTCTTTTCGCTATTCTTTTTATGATCTATACTATTGTTAGATACATTCATTTATGAAGGTGGCAAAATGGGTATGATTATTTAACGA
AATATATTAAAATAATTAGGTCTAATATTGTATAACAAATTCACCTCGTAATTATTTTTTATTTAACTACTCAAATACTTTTATTACTTTTTTAATTTTT
TTAACCAAATTAATAATTTTGAGTGTGTAAAATTTTAGTGCCAACACATATCATTTCTTATTCCTTTTGGATGATTTATTCTTCTCTTTTTTTTGACAAA
AAAAAAAAAAGATTTAGTGGGAGTTTTTTCTTAGGTTTGATCTTTTAAAATGATATATATTTGATATATATACGTAGAGAAAATAATAATTTTCTTAATA
AAATTTAGATGCATATAAATTTATAAATTTTTTTAGAATCGTGTCCGATAAATATTATTAATTTACATGACAAATGACATAACGGTCACGTAACGTTTAT
AAGTCAAAGTTTATTAATTTTGAATAAAATCATTAAATCAAAAAACAAAATAGAAAGTTAGGCCCTTCACTTCAAAAATTGAAATATAAAGAGGCAACAC
AAAAAAGAGAGAAGGGAAGATGAAAGAGTAATTTTGGAATTAAAGTACATTTCACTCGGCTATATATATATATATATGTATATGAATATGAATATATATA
TATATATATATAACAAAATACAATTAGAATACAAATTAGGCGCACCCAAAAGTCTCATCCACCATGACCATCCACTAGTCAGTTTTCAAAAAATGACATC
TCTAAATAGCAACCTCAAGCTTCATCCACTTTTCCCTTATATACAAATTGAAATATTTAATGAAGAGCTAATACCATAATGTTGCTACCTCAAAATAAAC
ACTAACCCATTTCCTTGTATATCATCAATGGACATTTTTTTCTACTTCTTCTCATATACAAAAGATTACCCACAAAAAGACATACATATAGTACCAGCAT
ATATACATATGCAACTATATATGTGTGTGTGTGTGTGTGTGTAAAAGAGAAAAATAATATAAGTTTTTGTGAGTGTGTGTGAGCTGGGTGATTTGCTGAA
GGGTCTATTTTTTGGTCTATAAATACCCATCTGCTAAGACAACCAACTTCTATTTTCTGCAAGTGCGTTATTACAAAGGAAGAGAGAGTGGGTTTTCAAG
AATTCTCATCCTTTCTTTCTTTCTCCCCTTCATCTTTTTCCATTAAGCTTAGCTAGTTTCCTTACCAGGTTAGTGGGTTTAATCCAAAAACCACACGTAA
CCCAAATAATAATTCCATTTCATTATGATATATACAAACACACACATATATGTATGTACTGATTCTGATCTATGTATGTTCTGGTTAAAACTTGACCATC
TGATCAAATCAAATCGTATGTTTTGGCTTTTCACCATTTTCAGCTTTCAACCACGGAGTTCCCTAATCAAGAACCGGAAAGCAGCTCTTCCCAGAAGAAA

> evm.TU.supercontig_414.3(CpSHP1)

AAAGAAGCACAAAGTAAACAAGGTTGGACCACAAATAGTTCCAACCCAATTAAAAAAGTTATTTATTAGAGATCTAAATTAACCAGGCAGAGATAAAATC
AGAATTACCAACTACGGTAATTCACAATATTTACAGCAACCTGATTCTCTCAGTGACTCATCTTTCTATTTCTATTTCAAGACATTGCAAAATATCTCTG
TTTACAAAATTTACAAACTTTAATTTATATGAATGCTTCTTAATTATGGCTATTAAGATTCTCTTCCCAAAAGTTACTGTTTCATTTTCTTATAGTTTTG
TATATATATATATTTTTTTGAGTCTATTTATTTAAAATTTTTAAATATAAAAATAAAATTTAAATTATGCATGAAATTTATAATCTGAAAAATAATTCAT
TAAAATATTATCTAGCAAACACTTTATAAAATATACTTTATTAAACTCTAAGTCTAAATTATAAACCAACACTAATATGAATAAGTATTACATAATCAAC
AAGAATTTAGTTTGCTTTCAATTAGTTGGAAAAAGTAATAAGTTTATTATCTAGCTATAATTTTTTTTTTGAATATAAATATATATGATTATAATATACA
TAAATATCTACTTGAAACTATATATACATAAATAATATATATATATATATAGCATGACCTGTCAAATGTGTGATGTCATGAGATTTTCTACATATATTGG
GCATGTTCAAGGAATTTGGCATCCTCACAACCTTTGAAAATTATATTTACCCCTTCCTAAAGGGATTTACGGACTCAATATTATTATATTTAATTAACAA
TAACAAAATCAATAAAATTAAAACGAATAATAATTTCATAAACTATGATAAATCATCTAACCCATAACTAATAAAATATAAAATATAATCCTCTCACGCC
TATTAATAATTACCTGACTTCATACTAAACTTTTGTTTTAAAAAATAAATAATTACTAGACATTAGTTTCAATTTTAATTTTAATTTTAATGGGTTTCAA
ATTTCGAAACGACAGGAGCGTGCTCTCACGCGCTCCAGTTCGCGGGTGGGAATGGGACATTCATGGCCGAGAAAGTGAAGGCAAACCACCCTAGACGGCT
AGAACACCCCACCCAGTTTGTTGCCAAAATTAAAATGGATAAAGAGAACAAGCGAAAGAAAAATAAACAACACTGAGTTTCCATTTTCTTGTAAGCGAAG
ATCCAGTGTAGTTTCTCTGTTGCGCGTAATATACACACACACACACACACACACACACACATATATCTATGTATTTATTTATATATGTATATATATATGG
AGCATCTGTAACTCCCACTTCTTGCTTCTCATATTAAGAGCCAAAACCTCAAATTACTATTACCATTAGACACAACCAACCACGGCTGACTCACCAATAC
AAGAATATATGTGCATATATAAAGGCATATAGACGTGTATATCTGTAGATATTTTCTGCATCTAATAGTTGGAAGGAAATTAAGAGACATTAAGGGGAAA

> evm.TU.supercontig_14.222(CpSEP1)

GGGGATAAAATTAAAGTCATTATCTGTCCTTCTTAGTAGAAACACACAACAAACAAGATTACCTCAACATTTATATAATCCTATAAAGGATCTTTCTTTT
TTAACATCACTAATTGGCTTCCCCATTTAGAAAAAAAACATTGACTATGATAAGTGTTTACATAACATGGTGAAAAAAATTTTCCACTACAAGGGTTAGG
AACTAACCCCACTATAGTTTATAAAGTAAATAAAATTGTTCCACTATAATTTTTTTCTTTTTATATCAACAAAATAGAAGCAAATAATATTAACAAAAGT
TTATTATTGATGAAGTTTCATATTTCCGGCAAATAATATTGTAATTAGTATAATATCATTTGTTACAAAAATTTTATCACCGACTTATATACTTCTAGCG
ACAATAGAGAAAGTATTACAATTATTTTTGATGACCCGCTTTTTGATGAAACAAAGTTTTGTCACTATATATATGTGAAATTTTTTCTTATTTACAATGA
AAAAATTTTCTATTAAAATTTTTACAGTCACTAGGTGTATTATTATTATCAGTAGTATTGAAAGGAGAACTCTAAGGCTTGTTGACAATTTTTGGTGAAT
GCCTAAGGCAAAAACTAAAGAAGTATGTTAAGCTAGTACAGGTCAAGCTAATATCAAATTTTAGTGCTAGAATTAACAAATTATGTCGAGGTTGACTAAC
GTGTGGAATGAACTAATTTAATTCAGGCTTGAAATCAAATTATTCGCTAAAGTTAGTTTTAGTAAAAGAAAAAACCAAGTGAACTTGGATTGGATTCGAA
ATTGGTTTAACAAATGTATTTGTTTTCAGTTGTAAAGCTCAAAATGGTAGTGAAAACCGCTAATTCTATGGATAAGTTAGGTTTAAAAGGATACTAAAAT
ATTAAAAAAAAATTGCACCATATATTAATTTGGTTTCAAACCAAATGAAGTCAATAATTTATTAATAATTTGTATGTTTCTAATACTTATATTTTTATAT
TTTATTTAAAATAAATTATTTCTTAAACTATTATCATAAGTGGGTATGATTGATTAGTCATAAAAATACACAAGATGTAGTAACTATCATAAAAAAAAAA
TAAATTAAATTATGAGAATTTACTGTTAAATTGTTTTATTACAATAAAAGTATGAAAGAAGTATTCAAGATATATTAGTAAAATGCTACATATTTATAAT
TCTTAACCTATAATAAAATTTATTTCTTAAACTTGTAGGATTAATGATTCAAGTCAAACCAATACATATGTACTTAATACTATCCTAGAGAAAGTTAATA
AAAATATTATTGTAAAAATATGAAAAAAAAAAAATTATGTGAGATTAACCCTAATTGAAAGTTTGATGGAGCATGGAGACACTTTCAGATATCTTTCCCT
AAATCATTGAAGGTTTTCATCCAATGGTTGACGAACACGTGGAGATGCCTTTTTAAGGTAGGTGACGACGTTCTGATGCTTCCGAGGACAACTAGAAATA

> evm.TU.supercontig_43.78(CpSEP2)

TACAACAACTAATAAATACATACACACATAATATATATATATATATATATAAAGCTAATCCCACAATCAATCTACCCTTTTATTAGCATATTCTGGATAA
AACTACAACACACACACACACACATTATAATTCAAATAAAAGTAATTATAATGTTAAAACCGTATATTAAGAACTCAGATTTGTCTATACAAATTTGGGT
AACAGCATGTTGTGTTTTAATCAGAGACCAAAGGAAAACCGTGAACCGGTGAATATGAACCTATTCAGGACGGTTCATGCTTTTTTAATCTGAACCGGTT
TGATTGGATTAGGTCCGGAGAGAGTTGAGTTAGGTCATATTTTAATGCACCAAACCCTATATTATGTACATTAATAAATTAGTCAACTATACCTAATATA
TGTAAATATATGTTGTTGGTTCATGCAATGGAATTACGGGAAGTCATTCTCGTACATGGGAGAGCATGGGATCAATTACTTTATATACATACATATATAT
ATACACACACACATTACTTAATTATAAAAATAAATTTTGACTTTTAATTTTTTAGTAATAAATAATGAAGAATTTATTTGGGAAATAAAAATTCAGAATC
TAGTTACAGAAATTAAAATTTTACTGACCAAATTGAATAAAACGTCAATTACCTTTTTACAATTAAATTTATTTATTATTTTTTTAAAGACAGGAGAGGA
GGAGTGACGGCAATTGTTTCATAACGGAACGGGGATAGATGGTTTAGAGTCGAGAGTCCCGTCGGAGAGAGGTTGAAGAAATGAGGGGCAATTCCGTAAA
TAGAGAGAGAAAACGTACGAACAAGGTTCAGTTTCTTAAGGTTAGGTCACATGATTTGCCCCAACCCGAAAATTGTCAGGTGGATTCAGACGCCCTCCTC
ACGACTTCTCCTCTTGACACGTGTCCAATTCACTCGTTATATTCCCATTCCTCATTTTAAAACGCGCTCCCTCTCCTCCCTTTCTGACCGTCCGATCTTA
AATTCTATCTTCAGATCCTGACACGTGGCTCAACTAACAGATAACAGCCTGTCTAATCATCTCTTAATTAATCTGTGTTTTAAACAATTACTACTAACCC
ATTTCCTTAATATAATATTAATTTTAGAGGACGTACTAGAGAATTAATTAATACAGGATCATCATCATCTCTCTATATCTCTTCTTCCTTTTTTTATTAT
TCTTTATTTATATATATATATATATATACAGAATTAAAAAAATGGAAAATAAAATAAAATAACCTGAGATTCTGTTATTAGCTGATGAGTAGAGTAGATG
CATGTGTAATGTAGCGGAGAGTATAAATGTATTACGAAGAGAAAAATAAATAAATAAAAAGGAAAAAAAAGAGAGGAAAAGCAAAAAAGAGAGATATATA
TGTTAAAATAGAGAGAACACAAAGGAATTGTGGATCGATTTGAAGTGTGAGAAAAGAGAAAAAAGGAAAAGAAAGAAAAAGAAAGAGAGAAAAAAAAAAA

> evm.TU.supercontig_660.1(CpSEP3)

ATATAGAGGGCGATGACAAAAACTACAACCGAAGAAAGTTTAAAAAATTTACATTGCAGATTCATTAATATTTTTATGATAATATATATGTTTTTCGAAT
ATGCCTAGTAGATAATTTGCAAATATATACATAATACTTATTGAGAATCATATAGACTATAAGTCATTAGAAAGCAAGAAAATGTGCCTAGTAGAAGGGA
TCTATTACATTTTTATTCATGTTATTACCTTTTTATCCCATGGATATATAAGTGTAGCTACTTCCACTATATGAAACATACTTTTTCTTTAAGGAACACT
ACATGAAAAACACTTTGATTCCCTCTAAAGAGCTCGATATGCTTGTGCTCTCAACATTTAACGTATCATGTTGGGAATGAAGCACATCTTGAACATAACA
ATTTGATTAATACTCTTCCAACATCTTATCCTCCATGTGATATTATGCGAATCATGATTATATATATATATATATATATATGCAAGACGTCGATCTATAA
AGAAAACATTAAATAAAAAGTGAGATAATAATTTAGTTAGACTTTCAAATCTTGTGAAGCAATATTTGATATTACAATGATTAGATTAGTCGAATGGAGC
ATAAGATTCAAAGACAGAGAAATGGAACCCTAAAAAGAAAGATTAAATTTTTAGCTTGTTGAAGAGTTTGACTAGGTCTCGTAGGTCCAGATTTTGGTCA
ACAATTAGATATTTCTTTTCTTTTAATACAGATGTTTAATTATATTTGTGACTATATAGGGGATATTATATATACTTACACATTTAGTTAAGATTAACAA
TGTTGAATTAACTGTTAATTGCATATTGAAACGAAGAAGATGCTTTTACTTTTCAAAGATGGCATTAATCTAGTGGAACCCTTTTTTTTTTCTTCTCTCT
TAAAAGCAAAGGATCTTAATTATTTATTTGAAAACACTTAAAAAAGCAAAAAAGATTAATGGAAAAAAAGAAACAAATTAATCTTTTCTTGAAAAATATT
TTAAATAAAATGAGGGTAATATATATAAATATTTATTTTGATAGTTGGTATATTTTAAAAAATGTTAAATTAAAAGTAAATGCAAAATGAAAGAAGAAAT
TCTGCAGATGACAGATGGTAATGCAATACAAGTAATATTCTTTTCCTGGCCTGGTATGAGAAAACACAGTAGCCAAGCCAAGGCAAAGGAGGGAGGAGAA
GAGAAACACAACGTACCCCACTTATCTTTCTCACTTTCTCTCTCTCTCTCTCTCGCTCTCTCTCTAGGTTAACCTGATACCTACTTACAAAAAGACGAAC
TCCTTTCTTGCCCTAATAAAAAAGAATCAGAGAATGCTCTACATCTACTCTACATTTTTATATATGCTCATTTATGGTCACTACCCAGAACATGTATTAC
TTCTCTTTTTGGGGTTAATATAAGTTGATAAGCAAAGCAAATACTATTACTATTACTACTTGTAACCACAAAAAAGCAAAAGAAAAGGAAAAGGGGAAAC

> evm.TU.supercontig_3.196(CpSEP4)

ACGGCGATGGCAACAAATAACTACCCACTATCATGACTTCATTCAATCCCATCTTGTCCCCTTCAATCAATAAAACAATTGGCATATACATACACACACA
CACACGCACGCATATATGTATATATTCTCATTAGAACATTATAATTGTCACGTGTTAAAAAAATCAAATGACCAAAAGAAAAGAAGGAAAGAGAACTTGG
TACGTTTATTAACCTGCGAACATAAATGACAAGAACTTGCGTTTAAAGATGGTGTGAGGTTTGAATATTTTTATTTTGTTAAATCAGTTTATGATATACG
TCGTGTTTGGTTATGGAGATATAATTTGACGTGTTTGGTTATGGAGATATAATTTGACGTGTTTGGTTATGAAAGGGATAGTCATGGAGGTGTTTGTGTG
ACCCATTTTATTTTATTAGTTAACTTATATATTTTCTTAGTCTTTACTAAAAAAATATAATTATTTTTTTGTTGTAATAAAATTTATTTTGTATTTTTTT
AATACATTTTTATAAAAATATTTTTATTAAATTTTGTTAATGTGTGTTTTTTTTTTGTATTTTTACCAATTTTTGCAAACGCTAATTATTTATAAAAGCT
TATACATTAAATAAAAATACAATACTCAATATATGTTTTACTTATTTTCAAGAATTTATAAAATTAATCTCATTTATTTCTAAATTTTATTTTTTGTATC
ATATGTTAATTCTGTATAATTAAATTGCGGCAATATATATATATATATATATAAATGATAAATTTGTACTGAGTAGAAATTTATCAATGAAACAAAAAAA
AAAAAATGAGTTTAATAATCTAGAGATTAACTCAAAAAGAATTTAGCTAAGAGGCCAGCAGTCCCTACATATCCTCCTCCTCCTTAAAATATGTCATATA
TATACATATACACAAACAGGATGATAGAAGTTTAATTAAATATATGTGCATATATATAGTAGGTAATCAGCAAATATTGAATATATACGTTTATATATAG
ACAGTAGACATATTTTCAACCTGTAACAATGTATGCAGTACTTTAGTTAATGATAAGTCTGATCAAAGAAAAGCATTAAAATACATGCATACAACTTTGG
ATGATACAGTGAAAAACCTTGGGTTTCTTTAACAGGTTAAGCCCTAAAACCAGACAAACAAATGTTCGATTTTTTGCTCTGCGTGTGTGTGTGTGTATAT
GTATAGACATATACATACACACCCATTAATCACACTCCCCCCCCAAAAAAAAGAAAAAGAAAAGAAAAAAAGGAAATGAGAGCATCTAAAACTTCTAATA
TAAACCACAGCATAAAAGCTCAAGATCTCACTCAAAACTCTTTCCATTTTTCTCTCTACCTATATTTTATCATTCCTCCATTAGTATAAATCCTTATTGG
TAAACCACAGATCGAGATTGTTCAAAAGTTCTTTCACCCTTTATCCAAGATTGGAAAAGAGAGTTAATCAGTGATCTTTTAGGTTTTTGTCAGAGGAATT

> evm.TU.supercontig_84.52(CpETR1)

ATGAACTCATTTATAGTTATGCAGATTTCCTAGCAGTTCCCTGTTGGATACCTTAGTGGTCAAGGGCAAAGCCTGGAAAACCCAACACCTTGGGTTCATT
TTCATGCTGCAAACTTGTGATTTAGAATGACTTGGAGCTAAGCATCACTCCTACAGTTTACCATGTATGGATGTGTCCAATACAGTGGTCAATAATTACC
AGGCATATGACAAGGAAAAAGTTCTTCCTGTTATAATTTAGCTTAGGTAACATGTTGTGTGTTCTGCCTTATAAGATTTAGTTATATTGTCTTTTAGATT
TTTTTTTTAGAGCTAAGAATTGAAATGTACCCATTTGGGTGCGATGGAGACAAGATTCTCCTCTTTATACCTGTGAGTACTTAAGGGACTGGTTGATGTT
TTGTCAGAGTTGTAAATAATTTTATACAGGTGGCACCCTCTTGGTGCTCATTAATACTATACTTTTTCCTTAAAAAAAGTAACATTTAATAGTTCAATCT
TGAACTAATGTTCTCAAAATGAATAGTAATGTTGCATATATCCATCCTTCATGTTTATTATGATACAAATTTATATAGAATTTGACGTGGTTGTGCTTAG
GATTTTTTAAAATCCAGCATACAAATCTATACAGAATTATTTATTTAATAAAAATTATAATATTTTATTTTACCTTACTGTCAAGAGCTTTGGACAGAGA
TCCTGATTTTAGGCTGAGATGAGAACTAGAGAGAGAATGAGACTTAAATGAGGATAGAAGTAGAGAATTCGAGATTACAAGAACACTCAGAAGTCAGACT
GATTTCAGGTAAACTGATAGCTTGTATTCAGTGGAGAATAAGGTTTGACATGTTTTTTCCTCCCTAATTCAGCCTATTTGTAGCTCAGTATAAGCTGTAA
CATTTATTCTGTTATGGAATTGCTACAATTTTTATTTGTACATGTGCTTCATGTGTCCTATCTCCTAAACCTGTTCCGCTTTGGTAAACCTTCAGCGGTT
TCTATCAATTTCGATGCTTTTTCATCTAAATACTTGTTTTATCTTTATAACATGAAAAATGTCTGTATGTATTTTACTGCTGCGGACAAAGTTTGAGATG
TGAGTTGAAGAAATATACTTCTTTTTTTTTTGTGCTTATACTTTTATTTTTATTTTGTATATATTTCCATTCTTGTTATTTAATTATTAATTATCAATTT
ATGCTGCTTTTATCTCAGGTTTGAATATCTGTTTGATTCTAAATGTGGATTTAAAGCTCAACTGAAAATTTAACATATACATAAAAAGCAGAGTAGGCAT
GCACCACAAGACAAGAACCAAACAAGAGTGGATGATCCTAGAAAAATTTAACTAAAATAATAAAAATCAGAAGTAAACAAGAGTAGATAAACGTGCTTTG
TATTTTCAATCAATTGTATGATCAAGAAAGGGAATTGCTGATTTATGTATCTTTGTCGTCAGGCGCTTGAAAGAGGAAGCACTGACTAGGTGGTTCAGTA

> evm.TU.supercontig_151.32(CpETR2)

AAATAATCCAATTTTAAATATATAAAATAAAATAAAATTATTACGATATATTATATAAAAAATAAATTACTTAATATAATATGTAACATTATGTTATCGT
GTTCTAGGACCAAATACTTTGTAACATTATGTTAAACTAGATTAGGTGAAATTCTAATTAAAGAAAAAAAATTCAAATTACTAACATCTGAATCCACCAT
TCTTGACCCTCAAAGTGGAAAGGTTAAAAAAAGAAAAAAGAAAAAAGAAAAAAAAGAAAAACCTTGGGTGTTCGCACGTGCGGCTAATTTTTTTAAATGA
CAGATTGATCGCACGTGCGAACGCCTTTGAAATCACTCGTCACTCGTTCCGTGTTGCCTTGGTGCGTGTCCGTGATTCTTTTGCCTCCCTCCCTCCACTT
TACTCCTTTTCTTTTTGTCTGCTCTTTACTGTTATTACTTACGTATTTCCCGGGTGGAAACAGCCAACGGAGCTCCCCGAGAGAGCTCTCGCGGCAAATG
GACGGCTCGGATCGCGTTCTCAAGCTCGCTGATTGATCTCTGCACGTAAGCTTCTTTATCTTCAAAGTTCTGCTTCAATGATTGATGTCTACGTGATGGT
TTTGGGTTCATTTTGAAATTTTCAGGTTTTCTGGAGGAGGATCAGATGCTTTCATTTGTTATTTGATCTTGTTTAATCATTTTTCAATATGTTCAAATGT
GTGGCGCGCTGTTTAGCCTAAGCTCGTTAAAGCTTGTTTGCTTAAGTGGAAAATTTTCCCGTGAGATTGTTTTCCTTTCTTTTTTAAAAGAAAAAGTTTG
ATTTATGCGTGCAGAATCTTGTATTGACCTTGGCTGAAGGTAACTGTGGAGAACTGATCCCGTTACTTCAGATTTTTTATGCAAGAGTCTAACCAAGCTA
CTGAGTGATACTTATGTGCTCCAGTGAGGTTCTTTCTTGATGATTAAGATGAAATTAATACGTCTTGGCTAGCATAAAGGCGATCTGAAAAATTTTAGAG
ATGTAATGAAACTAATTCGGACTATGTGTTGAGCATAGGTTCTTGCGGGAAGATATTAATGTTGTTGGTGGAGTAGGGGTTGACAGACTTGAAGCAACTT
TATTTCTATGTACTGAGTGAGGGAAGATATGGAGAAGAACGGAAGTGATAAGTTCTTTGATTTGGTATAACGAAGAATTTTACTTTAGTAAAGAATAGAA
GCCATTTTTGCAGGACTCCAAACTACTTTTGATGAATCTTTAACGTTTTGCTTCTATTAACCTTTCTGTGTCTGTCCATGGCTGCTAGTTCTTAATGAAA
TTGAATTTGCCATATATTTTTAAGAGACATAAACAAGTTATTGTCTTTTTGTTGGATTGGATATATCTTTCTTTGTTGAACACTGGGCATTTTGATTTCA
TTACCTCACTTTTTATGTGAGTATATTACACTGTTGCTTATAACTTATTTTTTCCTCTATATTGTCAGATTGACTGAGGCAGACAGGCAGTTGTCTGGAA

> evm.TU.supercontig_5.78(CpCTR1)

AAATCAGTTCAAACAGTTAACATAATAAAATAAAATAACATGTAATTTGAACAAAATATCATAAAATCATTATTTTTTCCTCACTGCCCCACATATACAT
ATGTAGATATCCTTATGAGTTGACTAAAAGTATATATTGGATCTTGTCCTTTCATGATCTCTTATTTAACTTATATTAATGCGTGCTTCCGATAAATTTG
ATTTGAGTTATCAACAAACTTTACACATAAGCTTATGTAATATTCATTCATAAGAATGGAACATAGCTTATTCAACGATATTAAAGTGATGGCAAATATA
TATGTAGATTCAAATCACTGAAGAAGATAACGTAATTGCCTTACATCATGGTTGTCCAAAATACCACAGTGTTGTCTTCGACAATTATCATATTGTTGTT
CAAATAGCTTTGTGTATAGTTTAACCATCGAGATAAGCTAATTGGGTTGGTGATTCATAAAATTTTAGATGAATTAATGTAAAAAAACATGAGAATACGT
TGAGTATGCCCGATTCTGACCAAATTAAATGAGAAATTATCAATAGAGTCCAACATAAGATATTAAAATGGAGAGTCTAATGAGTCTGGTGTAGCCTTGA
AGTTGCTGAAAAACTCGAGGGTGGCGGTGGTTACTTCAGTGCATGATCACAGTTATTTATAGGTATGAGAATGACTACGATTATTCTTATTATTTTTTAA
AATTGTGAATTGATTGAAATAAATTTATATTTATTTTTTTAAAAAATATTATACTAAATATCACGAGACATTAGACGTAACGATAGTAAGAATTAGTAAT
AAAAATAAATTAAATAATTTTTTTTCATAAATATAGGGATTAATATAATCAGAAATTAAATTGAGACTAGTTTTATATTTTAATAATTAGTTTTAAAATT
TCATAATTTGAAACAACTATTTAAAAATATAATATTTTTTTAAATACAATATAATAAAATATTAATCCTATTTCACCTCCATGAAAATATATATAAAATA
AAAAAGAGTAAAAAGGAAGAAAGTGGGAGTTGCGGTGCATGGAGTCATAAATAATCAACAAAGGCTGTTACGTGTAAATTCAGGTTTTCCTTCACCGGGA
AATGAATCAGCCAATACGCCTTCAAGGAGTGCAAAAAATCTCCAACCCATTCATCCAATCAACCCCTGAATAAAAAAAAAAAGTAAATACAAAAATCATT
TTATTAATCATTTAAAATACAAATATAAACTAAACATGCAGAAAATTACAATTTCGCACTCATAAACCCCACGNNNNNNNNNNNNNNNNNNNNNNNNNNN
NNNNNNNNNNNNNNNNNNNNNNNNCGTACTACGAGTCTCTATCCAGCGATAATAATAAGAGCGGTACCGCTACTTCTGCCAACAATAGCGGTAACAACAA
TAAAGTTAAAGCCGATAGAGGAGGATTTGATTGGGACCTCAACGCCGATCAGAGATTGAATCAGCAAACCAATCGGACTGGGAACTTATTTTCTTCCTCC

> evm.TU.supercontig_5.78(CpCTR1)

AAATCAGTTCAAACAGTTAACATAATAAAATAAAATAACATGTAATTTGAACAAAATATCATAAAATCATTATTTTTTCCTCACTGCCCCACATATACAT
ATGTAGATATCCTTATGAGTTGACTAAAAGTATATATTGGATCTTGTCCTTTCATGATCTCTTATTTAACTTATATTAATGCGTGCTTCCGATAAATTTG
ATTTGAGTTATCAACAAACTTTACACATAAGCTTATGTAATATTCATTCATAAGAATGGAACATAGCTTATTCAACGATATTAAAGTGATGGCAAATATA
TATGTAGATTCAAATCACTGAAGAAGATAACGTAATTGCCTTACATCATGGTTGTCCAAAATACCACAGTGTTGTCTTCGACAATTATCATATTGTTGTT
CAAATAGCTTTGTGTATAGTTTAACCATCGAGATAAGCTAATTGGGTTGGTGATTCATAAAATTTTAGATGAATTAATGTAAAAAAACATGAGAATACGT
TGAGTATGCCCGATTCTGACCAAATTAAATGAGAAATTATCAATAGAGTCCAACATAAGATATTAAAATGGAGAGTCTAATGAGTCTGGTGTAGCCTTGA
AGTTGCTGAAAAACTCGAGGGTGGCGGTGGTTACTTCAGTGCATGATCACAGTTATTTATAGGTATGAGAATGACTACGATTATTCTTATTATTTTTTAA
AATTGTGAATTGATTGAAATAAATTTATATTTATTTTTTTAAAAAATATTATACTAAATATCACGAGACATTAGACGTAACGATAGTAAGAATTAGTAAT
AAAAATAAATTAAATAATTTTTTTTCATAAATATAGGGATTAATATAATCAGAAATTAAATTGAGACTAGTTTTATATTTTAATAATTAGTTTTAAAATT
TCATAATTTGAAACAACTATTTAAAAATATAATATTTTTTTAAATACAATATAATAAAATATTAATCCTATTTCACCTCCATGAAAATATATATAAAATA
AAAAAGAGTAAAAAGGAAGAAAGTGGGAGTTGCGGTGCATGGAGTCATAAATAATCAACAAAGGCTGTTACGTGTAAATTCAGGTTTTCCTTCACCGGGA
AATGAATCAGCCAATACGCCTTCAAGGAGTGCAAAAAATCTCCAACCCATTCATCCAATCAACCCCTGAATAAAAAAAAAAAGTAAATACAAAAATCATT
TTATTAATCATTTAAAATACAAATATAAACTAAACATGCAGAAAATTACAATTTCGCACTCATAAACCCCACGNNNNNNNNNNNNNNNNNNNNNNNNNNN
NNNNNNNNNNNNNNNNNNNNNNNNCGTACTACGAGTCTCTATCCAGCGATAATAATAAGAGCGGTACCGCTACTTCTGCCAACAATAGCGGTAACAACAA
TAAAGTTAAAGCCGATAGAGGAGGATTTGATTGGGACCTCAACGCCGATCAGAGATTGAATCAGCAAACCAATCGGACTGGGAACTTATTTTCTTCCTCC

> evm.TU.supercontig_128.50(CpCTR2)

GAAGAGTAGCGACAGGAGAGAGGTAGAAGAAGAGTCCAGTGAGACTGTGAGATAAAAGATGGACAGGCTGCTTTACAACGAGGAAGTTGCAGTGGCTTGG
AGAAGGAAAGAATATCCAATTCACTGCTCTGCATACTTAAGTACAAATATGGAGAAGAAACACGACTTTACCTGGCTTAATCCAATGGAGATGGAACCGC
TAAGCAGTGAACCCCTGTAATTGTGTCCTTGATCGCAGGTTGAAGAAGAAGAAGAAGAAGAAGAAGAAGAAGAAGACAGGCTTTCCGCGGCGAGAAGAAC
GGAGCTTCGGTCTCCACCGCCGCACTCTCCTTTCTATTATTTCTTCGGATTTTGATGGGCCTGCGTTAATTACAAATGGGCTTTTTATTAAAATTAGCTG
TAAATTAATTAATTATTAATTAATTTTTCATTTTAATTTAGTAAATAAATTTATGAATTTTTTTTTTTAAAATATTTTTTAAAATATTTTTTTAATCTCT
ATTTTTAATAATATGAAAAATTATTACTTACCGAATTTTAATATTATAATTTATAATATATTTATTTTTTAATTAAATTTAAATTCATAATTTTTAAATA
AAATAAAAAAATTTCAATCATTAAATTATTATATTATTTTTTTATCAAACATAAAATACAACTAAATAAATTAATAATTTAATAATAATAATGACAATAA
ATCTCCCATCATATGAAAAGAAAATGACAACTAAATTAAATAAAAATTTCTCCAAATTTTGACATCCACAACTTTATAAAGTGTTATATTAACACATTAA
TTTATTTATATATTATTTAATATATATTTATTTACCTAAATAAAAATTAAAAAAAGTAAATATATATGTCAAAACACGTGTGCGAAATAACGTAATAATA
ATATTACAAAATAAAAAAAAATCATAAAATTCACAAGCAATTTTTTTTTTTCTCCATTCAAAATTACACGTGGACCCAGCTAATCTCCTTTAAGTCGAAT
TTGCGGAGAAGACTGAGTCGTAGGTTAAAAAAGTTGACATTATCGACGGTTCAGATTATTCGTCTGGATGAACTCAATTGTAGCGGATCATTTCTCCTGT
TCACTGAATCTTGAGTGCTTTCCTAGTTTATTTCTGGGACCCACAAAGCGTCGTTGTTGGTAAACGCAACCAAATGAAAAAGGAAAGCATCATTCTTCCT
GTCAAACTTTCAGGTCCCTCCATAAAAAAAAGCCACGAAAGCTCCATGAGTCGACCAAGTTCTCTGCTTGTTATGTAAGCTGTAACCAAAGAACAACTTC
TTTTCTTTCTATCATTTCCGAAATGACCGAACTTTGTTATCTCTTCCTCTGTTTACCCAATCTAAATACCCATCATAAAAGCTGAAACTGGAACTGAAAC
TTACAGAATTGTTTCTCTTTCTTTCACGAAGGGATAGAGAAGGTTATTATGTTGTGATCACCGGTGTGATAAGAGCTCTTCTTTCTGATTCCTGCAAGCT

> evm.TU.supercontig_9.380(CpACS1)

AGTGAATATTTTAATAAAAAAATAATTTTCACCAAAAACCAAAATTAATTATTGAAAATATAAAATAATAAAAATAGAGATTTGAGAGTTTTAAATTAAT
TTTTATATATATAACACTGTTATTATTATTTTGAATCATTAATTAATTTATATTTTTTGATTTCAATATATATAGCACGTGGTAGGTTGGCACGTTTTAC
ATTTGGTTAAATATTATAATTAATATAGAATTATGTAATATTTGTCTCGTCATTTGCAAAATAAATAATATAATAATTTTAATTGGTCATACCCTATGCA
TTTTTTGTTATAAATTTAACAATCAAAATAATATAAAACTATATTATATTCTTATTATGTATCAAAATCAAGACAAAAATAAGTAATTTTAGCCATTCTA
TATCCAAATAATTCAATATAAATTATGTATTTTTTTAAAAAAAAAATTGTTAGGAATTATAGGGATTTAATACAGACCATATTATATATCTAGATACATA
AATAACTTTGTATCAAGGGTTATGAATGCTGACAATAGTTTTTTTAATAAATATTATAATTTAATTGTACCATAAATACGGTAAGTCATTGTCTATAAAT
TATACATTCATAGCACACCAATTGATATAATAATTTGTAATAAATAAAACTATAGATTATAATTATCTTATTATAATAGAGGAATAAAAAAAGTACAAAC
TTTTGAATTTAAAAGCTATATAACTTGATAATATCGTTGCCTCGTTACTAAGTACATTCCCAACTTGATTATAATTTGTAAACAATTAATAAAAAATAAT
TGAATCAAATATTTAATATAATTTTTAATAATTTTAAATTTATCAAATCAATAAATGTTAAAAATAATTATAATTATTATCGAACCGTAAATTTTATTAA
TATATTGATTAAATATATGAAAAACTTACACAATCATAAATAATAAAAATTATATAATGAGATAAATTCAGAAAACTAAAAAGTCAATAAGTTTAGTAAT
ATAATTATTTTTTTAATAAATTATATAATTATAGAATTCAAAGGCTACGACTATAATTATATTTTGAATTGCTAACCATTATTATATTACAAGTTTAATT
ATTAGCAAAAATAAATAACATAATGAATTACATGAAGTTAATTTTCATGGCATTTATTATTATTATTATTAATTTTACGTGAATAAAAATAATAAATAAA
TATTATAATTTAATGGTAAAGATATTTTTTATATGGCCTCCAATTTATCATATTATTATTATTATTATTTTACTTTTAAAACTGTCGTTTCGGTCACTAA
AAGGTCATGTTGATGAAAAAAAGAAAAATATATTTTTTAAAAATGCATACGAATTAAATAAAATACAATAAATGTATTTTTTGTGCCTAAAGAACCGATT
CCGCGTTTCCAGTCAAATTCATTATCGTACGCACCCGTGTAAAATCCAACGGATTGTGCAAAGCCAACTCTCTCATATCAAGAATTCCTGAGAACTAAAT

> evm.TU.supercontig_117.57(CpACS2)

TAAAATTGATTTAATTAGATATCAAATGTATAAATTATAATTAGTTTATAGATATCAAATAAATTTTTAAAAAATTGAATCAGAATGAAAGAAATTTGAA
TAGAATGTAATGTAAATTCTGTCAAATACACAACATGAAAAAAGTACAAGAACAGTATCAATTTTAACACAATGTGAACCGGCCCTGGCACTATGCCAGG
GGCTTCAACCACATTGGACCGAAGAAAACAAAACTCCAATACACTTTGGCTTATATGCATACACATATATATACATACATTTTTGTGTGTTGACTTTTAA
AGGTATCATCTGTTACTAGATAATATTATCTTGTCATTTTGCCGAAATGAAAACCAGTTCATAACAAAACAAACGTTCATGTTTACACTTAATCAGTTCA
TTAATAGAAGTATCAAGTAGAAAGAAATAAAAGAAGCACTCTTGTTATAGTCGCAAAAAGCTTTTCAAGAGCAGCTAAAGTCCAGTTGCTCAATCTAACT
TGTACCAAGAAACCCAAGAAAATGGATAGGCGGAGATTACGTTAACAATGTTTGTATCAAAAAATTAGAGTGTAAACAATGCCATAGTTAATTCCAAAGA
CTTCTTTGTTCTGATCCAAGATAAGTCTTATCTGCTAAGCATGCATTTAAAAGTCTGACTGGTACTATCTTCTTGTCGTTCTACTGCTTGGTTTCTTGAT
ATAGAGAATAGATATAAGAGTTTCTATAAATATTCTTTGTTGTCTAGTTATCTAACTCTAATTCCCGGATACTTTCCGCTGCTGGTTGGTTCGAGGACTG
ATTCTTTTCCATTTTATGTTTTTTGCTCAGTTTGTGTTTGTTGCTTTGTCAGACTAAGTACTTCTTTTAGGGTATCTTCAATTGGTTTGGTGAAAGAAAA
TAAGCTTTAAGGGGTGATTCAATTTGCTTTTTTTTTTTTTTCACATGGGTATCAACTGATTTAGCTAGTAAAATATTAACATCTGATGATCTCACGAGGA
CTTAATCTGTCTATCTATATATATTCTGCTGATATTGATAGGAGTAACATTAGACAGCTGTCTGTGATTACAATTTTGCAGGATGCCATTTTGCTGATTG
CATGGTGATGATTGGCCATATGTTTTATTACAGTTGTTGGCTCTAGATGCTTTGGATTTTAGATGTTTCCATGTATGTAACTTTAGTGCTTGGTTTTGAC
TCCTCAAAATCTAACAGAACTAGAAATTTCCATCTGTATATTTCTCTTTTTGAGATGTCTGTCAACAGTCTTTTGTAATTCATACACGGTTTTAAATTTT
CACCTCTTATGCTCCTTGTAGTTCTTCTAGAAAGATAGCATGGAGGACACTTGCCGAATTAAAAAAGATGTTACAGAAGTAATTCATTCCCCAATACAAA
GCTTCTTTTATAGTAGTTATACTTGAATATTATCTTATTCATGTTCTTAGCCGTTTACTGGTTTCTATGTTGGTCTATACAGTTGATAGGAAATACCCCA

> evm.TU.supercontig_132.27(CpACO1)

ACCTACCGACCTCTTTTGCCTGACTGATAATATACGTTAATTAATTAAGGTTTTTTAATTAATTGTTTAATCAGGCAACCAATCTGTGTAATAGCTGATT
AACTTATATATTATGAACGATGAGGAGGAGGATTAAGTAGAGTTTGATTGGGAGAGGAACCCAGCAATAATTAACGTTTATAATTATTTAGAGAGATTAA
TTACTGTCTCTCCGCTGCGAGGCAATTAGTATGAAAATGGCATGTCGCCTAAATATCAACATAATTAAAATATTATTCAACGGTTCTTATTTAATTTTAA
TATAATATGTATGTACATTAAAAGCCAAAAGTTGCGATTATAAATGGAATCTAGAGCCAAAGTTTATTAATTAATTATACTGCTTGATTTAAGTTGTCGG
TCCTACATCAGATTTGCTGCTTCTGGGGAAAAAAAGTGTGAAAAAAAAAGTGCTAGAGTTATTATCTTTTCACCCACCAAATATATTAATAATAATTTTC
TTGGAAATAAAAAGATTAATGACGTTAAAAAATCATATATTTTTTTTTTGGGAATATTCTTATTGATTATTTTAATTCGTTTAAAACTTTTATTAAATTA
TGACGACATTATTTATAGTAATGTGTCAGGAATATTCATTATTTCCAGAATTAAACGAGAATGGCGAAGGGCATTCTAGTAATTATAGGGAAATCGTGCT
GTCAAATATAACGTTCAAAGCGTACGTATCGCACTTCGCAGCCCCACGTGCAAAACGTATTTATATATATATATATATATATATAGTACATTAGCCATAA
AATTATTTTTCAAGCAGCATTAAACATTGAAATAAATAAATAATAAACCATAAGTATTTATCGGATACATTTTATTAATTAATGTGTGTAGATTCTTATA
CAATAATTTGAAAAACTTCATTTAATTATTTTCTAATTAATAAATTAGATTACTACATTTTTAATTTGCTTTAAAAGTGTCTGCGTCCTTCATCAATTAA
TAATCATGCACGTAACCAGTTGGAAAGAAATGGACGGTCCATGATCGGCGGACTAGATACCGTCAAAACCACATTGCAGCACAGTCACTCGGGCAGTATC
TAAGCACCTTCCACCTATTGGTTTACTGGGGCCCCCATTCAGTAAAACGACCCAATAAGTCACGTAATTTAACGGTCTTCGTCTCCTGGGTCGGGTCAAC
AAATTAGACTCTATTATTTTCTTTTTAATTTTCAGTTAATCTTTGATATATATATATATTTTTTTTTTGAAAAATAATTTATTTATTTATTTATTTTTAT
GAGACTCCAAAACCCACTTCCACTTGCCTTCGCGGGCTACTCCTATTTCTGTCTTCATTCTGGATCAACCTCTATAAATTGCTTATCAAATCTCTCGAGT
CTGCATTCATAAACAACACCAAACACATCAAAAACTTTATTTACAAACAAACCCAGAAAAAGTTTCGACGATTTCTTCCCGAGAAATTCTTTCTGCAGAG

> evm.TU.supercontig_64.148(CpACO2)

ATGGACCTTTGTTTAATTTAATTTAATCTAATCTAATCACAGCTCTGCTCTGAGCGAAAGTGATAATTAAAAACCCTAATTTTTTTTTTTTTCTAGTTAG
GTTGAATATGCTGTTTGACTTTATTCATAGTTTAAAAGTCAAAATAACATTATAAATAATTAAGTTGAAAACAGCTTTGATTATCTATAATTTCTCACCC
ACATAATTAATTAACTTTTTTCTAATGCGAAAGGGATTAGGAGACTGGCCCGTTAATTATATACATATAATGATTAAATTAGACAATAATAATGGTCTTA
ATGCATGGAAAATCACATGCATCATCCCACATGATTCACCTCCAAGAACATGCCAATTGTGCAAGCAGAGTCTGCGGGCTGTATCAGCTGTTGAGAAGTA
CCCGGAAGTACATCAGAGGGTTTAATATATAATCAAATTTGTACCCTAACATTCCAACATTTGGAATCCAAATCACAACTGACACCGATACATGTAACTG
CATGTACAGCTAGCGCACCCTCTTGGGTTACAGATCATTTCCTTACTAGTTTGTATGTATGTATGTATGTATGTATGTATATACTGGAAGTTAACTGATT
GGTTTTGGGTAAGTTTCTAATATACTAAAAAGTAAAAGTATGAAGTTGACAATCCAATAATATAACTGGTTAATAAATTGAGCTGAAAAGTTGATCATGG
CTTTGTTTGTGCTTTACACCTCAACCCTGAAACTAAGCCTACAAATCAGTGTTTAGTACTTTAAATCATATGTTGACGATGACTAATCCATGTGTTGTGT
TAGAAGTCAAATGCTGATTAGGTAGTCAAAACCATTAACCCCAGTCGGCACAGATTAAAACTATTTCTTCACCCAAATGTAACAAGGAATTATATAGCTA
CTGTGTACTTCCAATAATTGTAAAGATACATTTGCTAAATGGGTATGAAAGTTAGAATTAGAATTCTATTTATTTGTAAAAAATCAATCAAAATAATATG
ATATTAATAAACGAATTTCGTTTTAATCGTTATCTGAGTCTTCGTCTATAATTAATGTTTATATTTTGATTCTTCTTTATTTTATTATGTTGTATTAGAA
AAATAATATGATATTATTTTATTATATACGACTAAAGTTAATAAACAAGAATAGAAATTTTGAAAAGACATGATCCCTCACATGGGTAAATTGTAAAATT
GATGTAGAGGTCGCCGTCCCATTCCTTTCCTTCACAGTTCTTCTTCTTCTTCTTCTTCTTTTCTAAAAAGAGAAAAAGAAGACAAATGTGTGACTGTGAC
TAACATTAATTAATTAATAAATTAAAGGGTTGACATATTTGAGGGTAGTGGTGGCAAATAGTATGATTATAAATAGCTCAAGTTAAAACACTTCTCATCC
CAACCCAACTATAGTCTTTACGTTCGCTAGTGTACCAGTCAATTTACAACTCCCTCATATATACACTTGTGTTCTTCTTGCTTCTTTACTTCTTTCATTC
