# Supplementary material for: Genome-wide identification and characterization of auxin response factor (ARF) family genes related to flower and fruit development in papaya (Carica papaya L.)
Source: BMC Genomics. 2015 Nov 5;16:901. doi: 10.1186/s12864-015-2182-0 (PMC4635992; doi:10.1186/s12864-015-2182-0)
Supplement: Additional file 13: Table S9. — The information of ARF family gene in Arabidopsis and rice. (DOCX 18 kb) [file 12864_2015_2182_MOESM13_ESM.docx]

| Species |  | |  | Species |  |  |
| --- | --- | --- | --- | --- | --- | --- |
| Arabidopsis | ID | | gene name | Rice | ID | gene name |
|  | AtARF1 | | AT1G59750.1 |  | OsARF1 | LOC_Os01g13520 |
|  | AtARF2 | | AT5G62000.1 |  | OsARF2 | LOC_Os01g48060 |
|  | AtARF3 | | AT2G33860.1 |  | OsARF3 | LOC_Os01g54990 |
|  | AtARF4 | | AT5G60450.1 |  | OsARF4 | LOC_Os01g70270 |
|  | AtARF5 | | AT1G19850.1 |  | OsARF5 | LOC_Os02g04810 |
|  | AtARF6 | | AT1G30330.2 |  | OsARF6 | LOC_Os02g06910 |
|  | AtARF7 | | AT5G20730.1 |  | OsARF7 | LOC_Os02g35140 |
|  | AtARF8 | | AT5G37020.1 |  | OsARF8 | LOC_Os02g41800 |
|  | AtARF9 | | AT4G23980.1 |  | OsARF9 | LOC_Os04g36054 |
|  | AtARF10 | | AT2G28350.1 |  | OsARF10 | LOC_Os04g43910 |
|  | AtARF11 | | AT2G46530.3 |  | OsARF11 | LOC_Os04g56850 |
|  | AtARF12 | | AT1G34310.1 |  | OsARF12 | LOC_Os04g57610 |
|  | AtARF13 | | AT1G34170.3 |  | OsARF13 | LOC_Os04g59430 |
|  | AtARF14 | | AT1G35540.1 |  | OsARF14 | LOC_Os05g43920 |
|  | AtARF15 | | AT1G35520.1 |  | OsARF15 | LOC_Os05g48870 |
|  | AtARF16 | | AT4G30080.1 |  | OsARF16 | LOC_Os06g09660 |
|  | AtARF17 | | AT1G77850.1 |  | OsARF17 | LOC_Os06g46410 |
|  | AtARF18 | | AT3G61830.1 |  | OsARF18 | LOC_Os06g47150 |
|  | AtARF19 | | AT1G19220.1 |  | OsARF19 | LOC_Os06g48950 |
|  | AtARF20 | | AT1G35240.1 |  | OsARF20 | LOC_Os07g08520 |
|  | AtARF21 | | AT1G34410.1 |  | OsARF21 | LOC_Os08g40900 |
|  | AtARF22 | | AT1G34390.1 |  | OsARF22 | LOC_Os10g33940 |
|  | AtARF23 | | AT1G43950.1 |  | OsARF23 | LOC_Os11g32110 |
|  | |  |  |  | OsARF24 | LOC_Os12g29520 |
|  | |  |  |  | OsARF25 | LOC_Os12g41950 |

**Table S9 The information of *ARF* family gene in *Arabidopsis* and Rice**

a. The information of *Arabidopsis* *ARF* genes was from <http://www.arabidopsis.org/>.

b. The information of Rice *ARF* genes was from <http://rice.plantbiology.msu.edu/>.
