# Supplementary material for: Genome-wide identification and characterization of auxin response factor (ARF) family genes related to flower and fruit development in papaya (Carica papaya L.)
Source: BMC Genomics. 2015 Nov 5;16:901. doi: 10.1186/s12864-015-2182-0 (PMC4635992; doi:10.1186/s12864-015-2182-0)
Supplement: Additional file 14: Table S10. — Primer sequences for qRT-PCR of CpARF family genes. (DOCX 17 kb) [file 12864_2015_2182_MOESM14_ESM.docx]

**Table S10 Primer sequences for qRT-PCR of CpARF family genes**

| Names | Sequences |
| --- | --- |
| CpARF1-RT-Up: | TGGATAATCACCCATCAGCA |
| CpARF1-RT-Dn: | TCACAGACCCATCAAACAGC |
| CpARF2-RT-Up: | ACGCCTTACACCAACACGAA |
| CpARF2-RT-Dn: | ACGCTGACTGCTTCCGACAC |
| CpARF3-RT-Up: | TTTAGGAATTTGCGAACAGG |
| CpARF3-RT-Dn: | CGAAGGCATTTGCTTACGAC |
| CpARF4-RT-Up: | GGTTGACCCACTGAGGAAGA |
| CpARF4-RT-Dn: | CGCCTGTTTAGCATGGAAGG |
| CpARF5-RT-Up: | AGCAACAGGGACTTGTAGGG |
| CpARF5-RT-Dn: | AGGGTTAGTGCATGGGAAAT |
| CpARF6-RT-Up: | CTGAATCACCAGCAACAAGC |
| CpARF6-RT-Dn: | CCAGCAGACAGCCAACAAAT |
| CpARF7-RT-Up: | TGGCTAAGGGAACGACAAAC |
| CpARF7-RT-Dn: | GCACCTGCTGACAACTGGAT |
| CpARF10-RT-Up: | CAGCCAGAGGAAGAAGAAGAC |
| CpARF10-RT-Dn: | CAGACGGTGATCGCAAAGGA |
| CpARF11-RT-Up: | TGAATTGTTGCCGGTTCATC |
| CpARF11-RT-Dn: | TTTGGGTGTCCTTGCTACTGC |
| CpARF16-RT-Up: | ATGATGGCACTGGGACTTGG |
| CpARF16-RT-Dn: | TGAGGACTCTTCGCGGATAA |
| CpARF17-RT-Up: | AGGTTGTCTGAAGCGGTGGT |
| CpARF17-RT-Dn: | ATAGTGAAGGCGGAGGTGGTG |
| CpActin1-RT-UP: | GAATGCATGTGAGCGATGAG |
| CpActin1-RT-DOWN: | TATCAGGTGACCAGGCAATG |
| CpActin2-RT-UP: | CTGTGCATGATGACTGCAAG |
| CpActin2-RT-DOWN: | TCATCTTGCTTCTCACCTTG |
